# Exome sequencing of early-onset patients supports genetic heterogeneity in colorectal cancer

**DOI:** 10.1038/s41598-021-90590-z

**Published:** 2021-05-27

**Authors:** C. Fernández-Rozadilla, M. Álvarez-Barona, I. Quintana, A. López-Novo, J. Amigo, J. M. Cameselle-Teijeiro, E. Roman, D. Gonzalez, X. Llor, L. Bujanda, X. Bessa, R. Jover, F. Balaguer, A. Castells, S. Castellví-Bel, G. Capellá, A. Carracedo, L. Valle, Clara Ruiz-Ponte

**Affiliations:** 1 Grupo de Medicina Xenómica (USC), Instituto de Investigación Sanitaria de Santiago (IDIS), Santiago de Compostela, Spain; 2 Hereditary Cancer Program, Catalan Institute of Oncology, Program in Molecular Mechanisms and Experimental Therapy in Oncology (Oncobell), IDIBELL, Barcelona, Spain; 3 Fundación Publica Galega de Medicina Xenómica, SERGAS, Grupo de Medicina Xenomica-USC, Instituto de Investigación Sanitaria de Santiago (IDIS), Santiago de Compostela, Spain; 4 Servicio de Anatomía Patológica, USC, Instituto de Investigación Sanitaria de Santiago (IDIS), Hospital Clínico Universitario, Santiago de Compostela, Spain; 5 Gastroenterology Department Hospital de Sant Pau, CIBERehd, Escola Universitària D’Infermeria EUI-Sant Pau, Universitat Autònoma de Barcelona (UAB), Barcelona, Spain; 6 Patología Digestiva, Hospital de Sant Pau, Barcelona, Spain; 7 Department of Medicine and Cancer Center, Yale University, New Haven, USA; 8 Hospital Universitario de Donostia, Instituto Biodonostia, Universidad del Pais Vasco (UPV/EHU), CIBEREHD, San Sebastián, Spain; 9 Gastroenterology Department, Hospital del Mar, Hospital del Mar Medical Research Institute (IMIM), Barcelona, Spain; 10 Servicio de Medicina Digestiva, Instituto de Investigación Biomédica (ISABIAL), Hospital General Universitario de Alicante, Alicante, Spain; 11 Gastroenterology Department, Institut D’Investigacions Biomèdiques August Pi I Sunyer (IDIBAPS), Centro de Investigación Biomédica en Red de Enfermedades Hepáticas Y Digestivas (CIBEREHD), Hospital Clínic, Universitat de Barcelona, Barcelona, Spain; 12 Hereditary Cancer Program, Program in Molecular Mechanisms and Experimental Therapy in Oncology (Oncobell), Centro de Investigación Biomédica en Red de Cáncer (CIBERONC), Catalan Institute of Oncology, IDIBELL, Hospitalet de Llobregat, Barcelona, Spain; 13 Fundación Publica Galega de Medicina Xenómica, SERGAS, Grupo de Medicina Xenómica-USC, Instituto de Investigación Sanitaria de Santiago (IDIS), Centro de Investigación Biomédica en Red de Enfermedades Raras (CIBERER), Santiago de Compostela, Spain

**Keywords:** Cancer, Genetics

## Abstract

Colorectal cancer (CRC) is a complex disease that can be caused by a spectrum of genetic variants ranging from low to high penetrance changes, that interact with the environment to determine which individuals will develop the disease. In this study, we sequenced 20 early-onset CRC patients to discover novel genetic variants that could be linked to the prompt disease development. Eight genes, *CHAD*, *CHD1L*, *ERCC6*, *IGTB7*, *PTPN13*, *SPATA20*, *TDG* and *TGS1,* were selected and re-sequenced in a further 304 early onset CRC patients to search for rare, high-impact variants. Although we found a recurring truncating variant in the *TDG* gene shared by two independent patients, the results obtained did not help consolidate any of the candidates as promising CRC predisposing genes. However, we found that potential risk alleles in our extended list of candidate variants have a tendency to appear at higher numbers in younger cases. This supports the idea that CRC onset may be oligogenic in nature and may show molecular heterogeneity. Further, larger and robust studies are thus needed to unravel the genetics behind early-onset CRC development, coupled with novel functional analyses and omic approaches that may offer complementary insight.

## Introduction

Heritability in colorectal cancer (CRC) is estimated to be between 8 and 40%^1,2^. However, only 5–10% is explained by rare, high-penetrance germline mutations in Mendelian susceptibility genes, such as those in Lynch syndrome—caused by pathogenic variants in mismatch repair (MMR) genes, adenomatous polyposis syndromes—caused by pathogenic variants in *APC, MUTYH, NTHL1* or in the exonuclease domain of *POLE* and *POLD1*^3,4^.

Except for the identification of *RPS20* as a causal gene for hereditary MMR-proficient nonpolyposis CRC, the studies undertaken in the past decade to identify new nonpolyposis CRC predisposing genes, have been mostly unsuccessful^5,6^. This suggests that the missing CRC heritability is presumably complex and polygenic in nature, and is caused by common, low-penetrance risk variants (such as those identified by genome-wide association studies), or by moderately penetrant rarer variants playing an important role in modulating neoplastic transformation^7,8^.

In this study, we performed whole-exome sequencing (WES) in 20 unrelated patients diagnosed with non-polyposis CRC at young age. Our purpose was to identify novel rare pathogenic variants that could explain such a premature occurrence of the disease.

## Results

### Patient description and sequencing

Whole-exome sequencing was performed on 20 early-onset CRC patients (< = 50 years). Median age at diagnosis was 45 years. All presented with MMR-proficient tumours, assessed as the conserved expression of the four MMR proteins MLH1, MSH2, MSH6 and PMS2. The clinical features of the patients are described in Supplementary Table 1.

Germline DNA obtained from peripheral blood was sequenced to achieve an average depth of 62.82X, where 77% of the targeted regions were covered by ≥ 10 reads. This provides a good assessment of inherited variant presence. After alignment, calling and annotation, a median of 76,122 variants were identified per individual (see Materials and Methods for a detailed description). No pathogenic variants were found in any of the known hereditary cancer genes that could account for the observed phenotype.

### Variant prioritization

Raw data were analyzed with a prioritization pipeline to reduce the number of candidate variants. Briefly, from the initial set of 337,011 unique variants, only those common to both the LifeScope™ (Thermo Fisher Scientific, MA, USA) and GATK calling algorithms were selected^9^, provided that they were found in the same patient and with the same genotype (n = 218,803). Variants annotated as synonymous or unknown were removed (n = 187,144), followed by restriction to exonic and splicing changes (n = 34,283). Common variants (MAF > 1% and > 0.1% for homozygous and heterozygous calls, respectively) were filtered out (n = 6,716 selected rare variants). Protein truncating—frameshift, nonsense -, variants at canonical splice sites, and missense changes predicted to have high-functional impact by at least 3 in silico predictors (Dann, Polyphen 2, CADD) and/or present at highly conserved positions (GERP +  +  ≥ 2), were selected (n = 358)^10–13^. Lastly, to exclude population frequency mismatches, we eliminated any variants found in a Spanish cohort of 267 non-cancer controls from the MPG cohort of the CIBERER Spanish Variant Server^14^. A total of 262 variants in 254 genes were finally selected after the implementation of this prioritization algorithm (Supplementary Table 2).

### Data analysis

#### Variant and gene-based analysis

**Figure 1:**
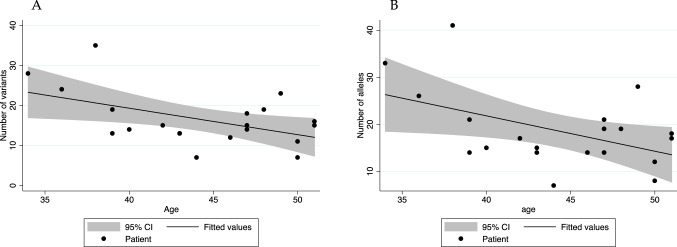
Relationship between number of candidate variants/alleles and age. Scatter plots depicting the significant trend between number of (**A**) risk variants (*p* = 0.021) and (**B**) risk alleles (*p* = 0.033) and age in the discovery cohort. Figure was created using Stata v11 (StataCorp—www.stata.com).

Considering this extended list of 254 candidate genes (Supplementary Table 2), there was a median number of 15 variants and 17.5 alleles per patient. We find a tendency for younger patients to have a higher number of rare, high-impact variants. This correlation is both true for the number of variants (*p* = 0.023) and the number of alleles (*p* = 0.033) (Fig. 1). Eight genes were found to have 2 candidate variants each after prioritization, and we found recurring variants, present in at least 3 individuals, in 11 genes The low presence of variants in recurring genes precluded us from doing gene-burden tests from the candidate gene list.

#### Pathway-based analysis

We performed pathway-based enrichment analyses based on KEGG pathways (https://www.genome.jp/kegg/) based on all 262 variants, to test whether there was an overrepresentation of potentially damaging rare variants in our cohort based on cancer-related pathways^15^. We focused on the following terms: Wnt signalling, TGF-β signalling, DNA repair, Pathways in cancer, Apoptosis, Cell adhesion molecules, Colorectal cancer, DNA replication, Hippo signalling pathway, MAPK signalling pathway, MicroRNAs in cancer, PI3K-Akt and PPAR signalling. However, none of the results were statistically significant after performing Fisher´s exact tests (Supplementary Table 3). We also performed hypothesis-free Gene Ontology enrichment analyses with PANTHER using Reactome pathway descriptions, also with no statistically significant results (Supplementary Table 4)^16^. Interestingly however, some of the top hit pathways correspond to known carcinogenetic routes, such as *TET1* demethylation, ion channel transport or glycogenolysis (as per the Warburg effect)^17^.

### Candidate gene selection

We selected 8 genes for replication in an independent familial/early-onset CRC cohort: *CHAD*, *CHD1L*, *ERCC6*, *ITGB7*, *PTPN13*, *SPATA20*, *TDG* and *TGS1* (Table 1)^18–21^. These were selected based on their prior description in early-onset sequencing studies, involvement on cancer-related KEGG pathways, presence of the mRNA/protein in healthy colonic mucosa (Protein Atlas, www.proteinatlas.org), or variants found in patients under 40 years (Supplementary Table 2).

**Table 1:** Candidate variants in the discovery cohort.

Gene	Variant description	Patient	Relevance
*CHAD*	NM_001267:c.1049C > T:p.(T350I)	PC-005;PC-015	PI3K-Akt pathway; present in individual with diagnosis before 40y; gene described in early-onset literature^16^
*CHD1L*	NM_004284:c.2398delT:p.(L800X)	PC-012	Loss-of-function variant; gene described in early-onset literature^17^
*ERCC6*	NM_000124:c.3437_3438insAAG:p.(S1146delinsRS)	PC-013	Nucleotide Excision Repair (NER); loss-of-function variant; gene described in early-onset literature^18^
*ITGB7*	NM_000889:c.1063_1066del:p.(V355fs)	PC-008	PI3K-Akt pathway; loss-of-function variant; gene described in early-onset literature^16,19^
*PTPN13*	NM_080685:c.4258 + 2 T > C	PC-008	Apoptosis; other gene family members have been described as potential candidates for CRC susceptibility^16^
*SPATA20*	NM_022827:c.151C > T:p.(R51X)	PC-015	Present in individual with diagnosis before 40y; loss-of-function variant; gene described in early-onset literature^16,19^
*TDG*	NM_003211:c.67C > T:p.(Q23X)	PC-018	Base Excision Repair (BER); loss-of-function variant; gene described in early-onset literature^17^
*TGS1*	NM_024831:c.439_444del:p.(147_148del)	PC-017	Peroxisome Proliferator-activated Receptors (PPAR) pathway; gene described in early-onset literature^17^

Potential risk genes identified in the discovery cohort and selected for replication, with their corresponding detected rare, high-impact changes.

### Candidate gene resequencing

#### Replication cohort

The replication cohort consisted of 304 non-related, MMR-proficient, familial and/or early-onset non-polyposis CRC patients recruited at the Hereditary Cancer Program of the Catalan Institute of Oncology, IDIBELL (Catalonia, Spain) (Supplementary Table 5)^22^.

Mutational screening of *CHAD*, *CHD1L*, *ERCC6*, *ITGB7*, *PTPN13*, *SPATA20*, *TDG* and *TGS1* was performed using a combination of PCR amplification in pooled DNAs and targeted massively parallel sequencing (See Materials and Methods for detailed description).

#### Candidate-gene variants

Prioritization filters were applied to the results of the targeted sequencing of the 8 genes, in an identical manner to those in the discovery phase. Thirteen variants in 6 of the candidate genes were found to fulfil our criteria. All variants were found in heterozygosity. None of the patients in the replication phase had more than one rare, high-impact variant in any of these genes (Table 2).

**Table 2:** Variants found in the eight candidate genes in the replication cohort.

Gene	Variant description	Age at diagnosis
*CHAD*	NM_001267:c.735C > A:p.(Y245X)	37 (patient I-0540-00) & 41 (patient I-2323-03)
*CHD1L*	NM_004284:c.607G > A:p.(G203R)	50
*CHD1L*	NM_004284:c.2273G > A:p.(R758Q)	50
*CHD1L*	NM_004284:c.2320G > A:p.(D774N)	39
*CHD1L*	NM_004284:c.263 T > G:p.(L88W)	45
*PTPN13*	NM_080685:c.1916G > A:p.(G639E)	49
*PTPN13*	NM_080685:c.2015A > G:p.(H672R)	50
*PTPN13*	NM_080685:c.5837G > A:p.(G1946E)	34
*SPATA20*	NM_022827:c.1177G > A:p.(G393R)	46
*SPATA20*	NM_022827:c.1426C > T:p.(R476W)	38
*TDG*	NM_003211:c.C67T:p.(Q23X)	49
*TDG*	NM_003211:c.T1175G:p.(I392S)	43
*TGS1*	NM_024831:c.107G > A:p.(R36Q)	42

Thirteen rare, high-impact variants were found in six of the eight genes selected for validation. None of the patients carried more than one variant.

Interestingly, the same variant in *TDG* (p.(Q23X)) was found in a single patient form the validation cohort as well as in the discovery cohort. We hence performed immunohistochemistry (IHC) assays on tumour biopsy samples from one of the patients to assess potential second allele inactivation. However, these revealed no differences in protein expression between the tumour tissue and the adjacent normal (Supplementary Figure S1).

Next, we compared the results found in our discovery and replication round with those found in the MPG Spanish reference cohort (control population)^14^. Twenty-three rare, high impact variants in 7 of the candidate genes were found in this cohort (Supplementary Table 5). Furthermore*,* we found no evidence for enrichment in rare variants in these 7 genes when comparing the cumulative allele count in the CRC patients with those in the MPG Spanish population reference dataset (Supplementary Table 6).

In parallel, we also looked into the prevalence of high-impact, rare variants in the eight candidate genes in the data published by Chubb and colleagues^19^. This included data on 1,006 early-onset familial CRC cases and 1609 healthy controls. Of the eight candidate genes, only *TDG* showed enrichment for nonsense, frameshift, missense predicted to be damaging and splice donor/acceptor-site variants in early-onset CRC patients compared to controls (n 8/1006 cases vs. 4/1609 controls) (Supplementary Table 7).

#### TCGA germline variants in the candidate genes

TCGA germline samples from colorectal cancer patients (from the COAD and READ cohorts; n = 219 Caucasians) were used to search for further evidence of variants in the 8 replicated genes being relevant to early-onset CRC^23^. Variants were assessed in the same way as described for the prioritization algorithm in “Variant prioritization” section. We found 21 heterozygous, rare, high-impact germline variants in 5 of the 8 replicated genes (Table 3). Seven of these were present in patients that had been diagnosed at 50 or earlier. We also observed a shift of variants in the complete list of genes in younger TCGA patients, although in this case, it was not statistically significant (*p* = 0.817) (Supplementary Fig. S2).

**Table 3:** Variants in the candidate genes in TCGA CRC samples.

Gene	Variant	Age at diagnosis
*CHD1L*	NM_004284:p.(M383I)	66
***CHD1L***	**NM_004284:p.(R468W)**	**49**
*ERCC6*	NM_000124:p.(L224F)	70
***ERCC6***	**NM_000124:p.(G601S)**	**46**
***ERCC6***	**NM_000124:p.(R683Q)**	**50**
***ERCC6***	**NM_000124:p.(F1437I)**	**43**
*ITGB7*	NM_000889:p.(Y753C)	60
*ITGB7*	NM_000889:p.(Y758fs)	76
*PTPN13*	NM_080685:p.(S348T)	75
*PTPN13*	NM_080685:p.(F724L)	89
*PTPN13*	NM_080685:p.(R782X)	60
*PTPN13*	NM_080685:p.(R817C)	74
*PTPN13*	NM_080685:p.(E1047G)	68
***PTPN13***	**NM_080685:p.(T1383M)**	**47**
*PTPN13*	NM_080685:p.(G1420R)	74
***PTPN13***	**NM_080685:p.(D2110G)**	**41**
*PTPN13*	NM_080685:p.(R2371H)	78
***PTPN13***	**NM_080685:p.(R2446H)**	**43**
*PTPN13*	NM_080685:p.(Q2482X)	73
*SPATA20*	NM_022827:p.(R51X)	77
*SPATA20*	NM_022827:p.(V596M)	78

Twenty-one variants in five of the candidate genes were found in TCGA CRC COAD/READ samples. Seven of these were found in patients under 50 years of age (in bold).

Tumour variation profiles were also inspected in the search for a second somatic hit in the TCGA patients carrying these mutations (https://portal.gdc.cancer.gov/). We found an additional somatic missense variant (p.(P677L)) in a patient carrying the germline *ERCC6* p.(R683Q) change. Because *ERCC6* is implicated in DNA nucleotide-excision repair (NER) repair, we inspected the mutation signature profile of the tumour with the help of the MuSiCa software^24^. It showed that signature 3 (associated with homologous recombination) was actually the most prevalent, whereas patterns related to NER (albeit not with CRC), such as signatures 4, 7, 11, 22 and 24, contributed only marginally, which did not support our hypothesis that the *ERCC6* mutations were the drivers behind the early development of the tumour (Supplementary Table 8) (https://cancer.sanger.ac.uk/cosmic/signatures_v2.tt).

Interestingly, for the remaining TCGA patients carrying germline changes in these candidate genes, over half of these mutation profiles showed a predominant effect of signature 3. This signature has been related to deficient repair processes involving *BRCA1* mutations but was not expected directly for a tumour arising from the variants in our candidate genes (Supplementary Table 8).

## Discussion

In this study, we performed whole-exome sequencing to discover novel rare CRC susceptibility variants that could be responsible for the early onset of disease observed in these patients. For this purpose, we selected candidate high-impact rare variants obtained from the sequencing of 20 unrelated early-onset CRC patients. Then, we selected 8 genes as potential candidates, to be replicated in a validation cohort.

Remarkably, *TDG* turned out to be the most interesting gene in our analyses. It is a BER gene that has been proposed as a tumour suppressor as well as a Wnt pathway regulator and epigenetic modifier in CRC^25–29^*.* In our study, a *TDG* truncating variant p.(Q23X) appeared in two unrelated individuals. Unfortunately, the IHC results did not show any differences between normal and tumour protein levels to account for a second somatic event. Hence, the results obtained for these 8 genes from our analyses did not provide support enough to claim any of them as a strong candidate for CRC susceptibility^19^.

The causes for this lack of conclusive results may be several. Firstly, the fact that all patients present with an early onset of the disease does not necessarily mean that the underlying genetic cause is homogeneous^4^. This is supported by the fact that we hardly found recurring variants or genes in our complete list of rare high-impact variants. If so, then larger discovery cohorts would be needed to assess this heterogeneity reliably.

Secondly, CRC development is likely due to arise from the interaction of multiple genetic variants plus environmental factors (i.e. it is oligogenic in nature)^30,31^. Interestingly, we found a trend that younger patients have a higher proportion of rare, high-impact variants and alleles. Moreover, we do observe that some of the top hits in our pathway analyses correspond to known CRC carcinogenetic pathways, including *TET1* methylation^32,33^, ion channel transport^34^ and colorectal carcinogenesis^35,36^. These would support the idea of multiple, moderately penetrant variants being responsible for the early-onset phenotype.

Lastly, the vast amount of data produced by sequencing makes it necessary to utilize prioritization algorithms that are somewhat arbitrary. The approach chosen for prioritization depends on prior knowledge of the patient selection criteria (as described above), the damaging effect of variants encountered in the genes, the inheritance model and the function of the genes affected, which is often (if not always) unavailable^37^. In this complicated scenario, it is then easy to envisage why not only this current study, but also other previous works inspecting the genetic contribution to early onset CRC development have been underwhelming. In this sense, other strategies may be pursued in order to explore the data more comprehensively. For instance, integration of genetic variation with other data sources such as transcriptomic gene expression of methylation levels may be useful in prioritizing candidates in a more meaningful way^38^.

In any case, it is guaranteed that larger studies are needed. These ought to be appropriately designed and powered to detect the expected genetic heterogeneity. In the era of Open Data Science, we must hence walk towards making coordinated efforts in order to obtain robust results, particularly for cases that are rare within the CRC spectrum, and that are presumably molecularly heterogeneous. Hopefully too, the near future may also facilitate data interpretation via complementation with functional data, such as CRISPR assays or in vitro organoid models, which would be certainly helpful to increase the throughput in the functional screening of candidate genes, and may prove invaluable in validating novel CRC susceptibility loci.

## Materials and methods

### Study patients: discovery cohort

The study received the approval of the Ethics Committee (CEIC *Comité Ético de Investigación Clínica de Galicia *(2011-123). The discovery dataset consisted of 20 non-related and unexplained MMR-proficient CRC cases from the EPICOLON consortium^39^. All patients received informed consent and protocols were at all times in accordance with the tenets of the declaration of Helsinki. The selected patients were all diagnosed with CRC before/at the age of 50, with tumours showing microsatellite stability and/or conserved expression of the MMR proteins (MLH1, MSH2, MSH6, and PMS2). Patients either showed apparently sporadic, autosomal recessive or incomplete-penetrance dominant inheritance patterns. None of these patients had detectable germline mutations (point mutations or small indels) in the MMR, *MUTYH* or *BMPR1A* genes, as assessed by bidirectional Sanger sequencing and Multiplex Ligation-dependent Probe Amplification (MLPA) initially, and verified by exome sequencing in this study.

### Whole-exome sequencing (WES)

WES was performed on genomic DNA extracted from peripheral blood cells of all patients. DNA extraction was undertaken using the CHEMAGEN robot (Chemagen Biopolymer-Technologie AG, Baesweiler, Germany) and the sequencing was performed using the SureSelect Human All Exon kit V5 for library preparation (Agilent Technologies, Santa Clara, CA, USA) and ran on a 5500xl SOLiD™ system (Thermo Fisher Scientific, Massachusetts, USA). The sequencing reads were aligned to the hg19 reference genome using the software provided by the sequencer. Variant calling was performed using both the Lifescope and GATK 3.0 suites. For LifeScope, variant QC was performed employing the diBayes parameter of low astringency level (dibayes.het.min.start.pos = 2 and dibayes.hom.min.nonref.start.pos = 2). Additionally, we removed variants with a depth < 30X, variants with a calculated strand bias p value > 0.05, and all alternative allele variants with < 4 reads to minimise artifacts and select for high quality variants. For GATK, variant QC comprised filtering by QUAL >  = 30, a minimum depth per variant of 30 and removal of variants with strand bias *p* < 0.05. An additional filter was applied to exclude variants with < 20% and/or < 4 variant reads, to mimic LifeScope stringency filters. Variants were annotated using ANNOVAR (version 2019Apr09).

### Variant prioritization

A prioritization pipeline was applied to reduce the number of variants of interest. Firstly, only variants common to both LifeScope and GATK calling were selected, given that they were found in the same patient with the same genotype. Later, rare variants (MAF_gnomAD2.1.1_NFE_ ≤ 0.1% and 1% were chosen for heterozygous and homozygous and calls, respectively). High-functional impact changes: loss-of-function (LoF) variants resulting in truncated proteins (nonsense, frameshift) and variants at canonical splice sites (+ -1/2) were selected, together with high-impact missense variants predicted by at least three in silico tools: PolyPhen-2 (selected if probably/possibly Damaging—D/P), *CADD_phred* ≥ *15, Dann* ≥ *0.995*), and/or located at conserved sites (GERP +  +  ≥ 2). Next, variants present in a representative population cohort of 267 control Spanish were eliminated to discard population ancestry bias. These belong to exome sequencing data from “non-cancer” healthy controls from the CIBERER Spanish Variant Server [ref same as above]. Sequencing artefacts were removed by curating using an in-house database of around 1,300 exomes produced with the same technologies. Variants in the eight candidate genes were validated by bidirectional Sanger sequencing.

#### Variant-based analysis

Frequency deviations between the detected in our cohort and the expected (as per gnomAD v2.1.1 counts in non-Finnish Europeans—NFE) were calculated for variants with at least 3 observed alleles using chi-squared tests of Fisher’s exact test if allele counts < 5.

### Gene and pathway-based analyses

We selected the following cancer-related KEGG pathways to test whether there was an enrichment of potentially damaging rare variants in our cohort: hsa04310 (WNT signalling), hsa04350 (TGF-β signalling), hsa03430 (MisMatch Repair, MMR), hsa03410 (Base-Excision Repair, BER), hsa03420 (Nucleotide Excision Repair, NER), hsa03440 (Homologous Recombination, HR), map03450 (Non-Homologous End Joining, NHEJ, NHJ), hsa03460 (Fanconi Anaemia), hsa05200 (Pathways in cancer), hsa04210 (Apoptosis), hsa04514 (Cell adhesion molecules), hsa05210 (Colorectal cancer), hsa03030 (DNA replication), hsa04390 (Hippo signalling pathway), hsa04010 (MAPK signalling pathway), hsa05206 (MicroRNAs in cancer), hsa04151 (PI3K-Akt)], hsa03320 (PPAR signalling). For this, we performed a Fisher’s exact test using R^40^. A nominal *p* value of 0.05 was considered significant.

### Gene prioritization and candidate-gene selecion for replication

We prioritized genes that had been described in previous works on early-onset CRC patients and/or those belonging to cancer KEGG pathways. Amongst those, we selected 8 genes for replication based on their involvement on cancer-related KEGG pathways (as described for the pathway analysis), presence of the mRNA/protein in healthy colonic mucosa (Protein Atlas, www.proteinatlas.org), genes with variants in patients under 40 years. All candidate variants in the 8 selected genes were validated using Sanger sequencing.

### Candidate-gene resequencing

#### Replication cohort

For the replication, a total of 304 non-related unexplained MMR-proficient early-onset non-polyposis CRC patients were included. All cases were affected with CRC. The mean age at cancer diagnosis was 41.73 (range: 16–50). A detailed description of the series has been described in Belhadj et al.^22^. All patients were of European origin, and were assessed at the Hereditary Cancer Program of the Catalan Institute of Oncology (Spain) between 1999 and 2017. The study received the approval of the Ethics Committee of the *Institut d’Investigació Biomèdica de Bellvitge* (IDIBELL) (PR247/15). As for the discovery cohort, all patients received informed consent and recruitment complied with the tenets of the declaration of Helsinki.

#### Candidate gene sequencing

Targeted resequencing of the 8 novel candidate CRC susceptibility genes was performed on genomic DNA extracted from peripheral blood cells extracted using the FlexiGene DNA kit (Qiagen, Valencia, CA). Mutational screening was performed using a combination of PCR amplification in pooled DNAs and targeted massively parallel sequencing^41^. Primers used for amplification were described in the original publication. DNA pools were obtained adding equimolecular quantities of each sample (# samples/pool: 48-96). The resulting pools were used as templates for PCR amplification of each region of interest using the Phusion High-Fidelity DNA Polymerase (New England Biolabs, Ipswich, MA, USA). PCR products were checked by electrophoresis, purified (QIAquick PCR purification Kit, Qiagen, Valencia, CA, USA) and quantified (NanoDropTM, Thermo Fisher Scientific, Waltham, MA, USA). Equimolecular amounts of each purified amplicon were pooled, ligated and fragmented using a Covaris S2 (Covaris, Inc. MS, USA). DNA libraries and next generation sequencing (NGS) at high coverage were performed on a HiSeq-2000 (Illumina, San Diego, CA, USA) at Centro Nacional de Análisis Genómico (CNAG, Barcelona, Spain). FASTQ files were mapped to the reference genome GRCh37/hg19 using the Burrows-Wheeler Aligner (BWA-MEM). BAM files were generated using SAMtools. Only the bases with base quality ≥ 30 were used for the analysis. Base-level metrics of all positions were extracted using Bam-Readcount. The generated data was used to calculate the estimated number of mutated alleles per pool (ENMA), which depends on the number of samples included in each DNA pool. All genomic positions were annotated with ANNOVAR and common variants present in the Genome Aggregation Database (gnomAD v.2.1.1) with a minor allele frequency (MAF) ≥ 1% were filtered out. The median number of reads per base obtained for all coding regions and + /− 5 bp flanking regions of the 8 genes analysed was 9421(5–31,562 reads/base). The selection of the high-impact functional rare variants was performed using a prioritization pipeline identical as described above for the discovery cohort.

#### Variant validation

Variant-specific KASP genotyping assays (LGC Genomics, Hoddesdon, UK) and/or direct automated (Sanger) sequencing were used to validate all the variants in the candidate genes. Sequencing was performed at STAB VIDA (Caparica, Portugal) and Macrogen (Amsterdam, the Netherlands) and data was analysed with SeqMan Pro (Lasergene 13, DNASTAR, Madison, WI). The primers used for amplification and sequencing were the same as the ones used to amplify the pooled DNAs.

### TCGA germline variants on candidate genes

TCGA germline variation was inspected for high-impact germline variant, as described in “Variant prioritization” section. For this, aligned bam files were obtained with permission access from the GDC data portal (https://portal.gdc.cancer.gov/). Germline variant calling was accomplished using VarScan2. This software is one of the callers used to generate somatic variation files, allows for the implementation of a germline calling pipeline as well. The results were filtered following the variant criteria described in the previous sections for colorectal COAD and READ samples. Additionally, results were restricted to samples of reported white ethnicity to account for population mismatches on allele frequencies.

#### TCGA second hit validation and somatic mutation profiles

VarScan2 annotated somatic vcf files were retrieved from the GDC data portal for the patients identified as carrying potential germline variants in the candidate genes, and analysed following the same variant selection criteria. Somatic mutation profiles were obtained using the MuSiCa software for mutational signatures using COSMIC v2 profiling.

### TDG immunohistochemistry

Immunohistochemical assays were carried out using 4-μm-thick paraffin sections in an automatic immunostainer (Autostainer Link 48; Agilent, Santa Clara, CA, USA) equipped with a 2-step immunohistochemical staining system (EnVision FLEX/HPR; Dako, Glostrup, Denmark) that uses a peroxidase-labelled polymer conjugated to the secondary antibody. Before the immunostainer, the samples were treated for antigenic retrieval according to the manufacturer’s protocol in the pre-treatment module (PT link, Dako; Agilent). Samples were incubated with a primary antibody against TDG (thymine-DNA glycosylase) (polyclonal, pH6, dilution: 1:100, 40 min, Sigma Life Science, St Louis, MO, USA). Nonimmune serum samples were substituted for the primary antibodies as negative controls. Normal colonic mucosa was used as a positive control.

## Conclusions

Sequencing of patients with early-onset CRC may be an important tool to discover novel susceptibility variants associated with an early-onset of the disease. In our study, we have discovered evidence that this early disease development may be the result of multiple and variable rare moderately-penetrant variants, i.e. CRC susceptibility is oligogenic and heterogeneous. Hence, further larger and appropriately powered studies are necessary in order to unveil the genetics behind it. Alternative complementary approaches such as using interrelated omic sources and high throughput functional studies may in the near future offer refinement on strategies based only on gene prioritization algorithms.

## Supplementary information

Supplementary Information.
