# Supplementary material for: Exome sequencing of early-onset patients supports genetic heterogeneity in colorectal cancer
Source: Sci Rep. 2021 May 27;11:11135. doi: 10.1038/s41598-021-90590-z (PMC8159954; doi:10.1038/s41598-021-90590-z)
Supplement: Supplementary file 1 — Supplementary Information. [file 41598_2021_90590_MOESM1_ESM.docx]

Supplementary Information

**Exome sequencing of early-onset patients supports genetic heterogeneity in colorectal cancer**

*Fernández-Rozadilla C^1†^, Álvarez-Barona M^1†^, Quintana I^2^, López-Novo A^1^, Amigo J^3^, Cameselle-Teijeiro JM^4^, Roman E^5^, Gonzalez D^6^, Llor X^7^, Bujanda L^8^, Bessa X^9^, Jover R^10^, Balaguer F^11^ Castells A^11^, Castellví-Bel S^11^, Capellá G^12^, Carracedo A^13^, Valle L^12^ and Clara Ruiz-Ponte*^13^*

^1^Grupo de Medicina Xenómica (USC); Instituto de Investigación Sanitaria de Santiago (IDIS); Santiago de Compostela, Spain; [Ceres.Fernandez.Rozadilla@sergas.es](mailto:Ceres.Fernandez.Rozadilla@sergas.es); [Miriam.Alvarez.Barona@sergas.es](mailto:Miriam.Alvarez.Barona@sergas.es); [anael.lopez@rai.usc.es](mailto:anael.lopez@rai.usc.es)

^2^ Hereditary Cancer Program, Catalan Institute of Oncology; Program in Molecular Mechanisms and Experimental Therapy in Oncology (Oncobell), IDIBELL; Barcelona, Spain. [iquintana@idibell.cat](mailto:iquintana@idibell.cat)

^3^ Fundación Publica Galega de Medicina Xenómica, SERGAS, Instituto de Investigación Sanitaria de Santiago (IDIS), Grupo de Medicina Xenomica-USC, Santiago de Compostela, Spain; [Jorge.amigo@usc.es](mailto:Jorge.amigo@usc.es)

^4^ Servicio de Anatomía Patológica, Hospital Clínico Universitario, Instituto de Investigación Sanitaria de Santiago (IDIS), USC. Santiago de Compostela, Spain; [josemanuel.cameselle@usc.es](mailto:josemanuel.cameselle@usc.es)

^5^ Escola Universitària d’Infermeria EUI-Sant Pau, Gastroenterology Department Hospital de Sant Pau, CIBERehd; Universitat Autònoma de Barcelona (UAB), Barcelona, Spain; [ERoman@santpau.cat](mailto:ERoman@santpau.cat)

^6^ Patología Digestiva, Hospital de Sant Pau, Barcelona, Spain [DGonzalezJ@santpau.cat](mailto:DGonzalezJ@santpau.cat)

^7^ Department of Medicine and Cancer Center, Yale University, USA; [xavier.llor@yale.edu](mailto:xavier.llor@yale.edu)

^8^ Hospital Universitario de Donostia, Instituto Biodonostia, Universidad del País Vasco (UPV/EHU), and CIBEREHD, San Sebastian, Spain; [LUIS.BUJANDA@osakidetza.net](mailto:LUIS.BUJANDA@osakidetza.net)

^9^ Gastroenterology Department, Hospital del Mar; Hospital del Mar Medical Research Institute (IMIM), Barcelona, Spain. [xbessa@psmar.cat](mailto:xbessa@psmar.cat)

^10^ Servicio de Medicina Digestiva. Hospital General Universitario de Alicante; Instituto de Investigación Biomédica (ISABIAL), Alicante, Spain; [Jover_rod@gva.es](mailto:Jover_rod@gva.es)

^11^ Gastroenterology Department, Institut d'Investigacions Biomèdiques August Pi i Sunyer (IDIBAPS), Centro de Investigación Biomédica en Red de Enfermedades Hepáticas y Digestivas (CIBEREHD), Hospital Clínic, Universitat de Barcelona, Barcelona, Spain; [FPRUNES@clinic.cat](mailto:FPRUNES@clinic.cat), [CASTELLS@clinic.cat](mailto:CASTELLS@clinic.cat), [SBEL@clinic.cat](mailto:SBEL@clinic.cat)

^12^ Hereditary Cancer Program, Catalan Institute of Oncology; Program in Molecular Mechanisms and Experimental Therapy in Oncology (Oncobell), IDIBELL; Centro de Investigación Biomédica en Red de Cáncer (CIBERONC); Hospitalet de Llobregat, Barcelona, Spain; [gcapella@idibell.cat](mailto:gcapella@idibell.cat); [lvalle@idibell.cat](mailto:lvalle@idibell.cat)

^13^ Fundación Publica Galega de Medicina Xenómica, SERGAS, Instituto de Investigación Sanitaria de Santiago (IDIS), Grupo de Medicina Xenómica-USC, Centro de Investigación Biomédica en Red de Enfermedades Raras (CIBERER), Santiago de Compostela, Spain; [angel.carracedo@usc.es](mailto:angel.carracedo@usc.es), ; , clara.ruiz.ponte@usc.es

^†^ These authors contributed equally to this work

***** Correspondence: [clara.ruiz.ponte@usc.es](mailto:clara.ruiz.ponte@usc.es)

**Supplementary Table 1. Clinical features of the discovery cohort.** The 20 selected samples are unrelated patients with no identifiable pathogenic variants in any of the described hereditary CRC genes that could explain the early-onset phenotype. NA: not available

| **Sample** | **Sex** | **Age at diagnosis** | **Tumor site** | **Family history of cancer** |
| --- | --- | --- | --- | --- |
| PC-001 | Female | 43 | Sigmoid colon | No |
| PC-002 | Male | 42 | NA | No |
| PC-003 | Male | 50 | NA | No |
| PC-004 | Female | 39 | Transverse colon | No |
| PC-005 | Male | 38 | Rectum | No |
| PC-006 | Male | 47 | Descending colon | Sister CRC, father leukaemia |
| PC-008 | Male | 48 | NA | No |
| PC-009 | Female | 50 | Sigmoid colon | No |
| PC-010 | Female | 44 | Rectum | No |
| PC-011 | Male | 36 | Rectum | No |
| PC-012 | Male | 50 | Sigmoid colon | No |
| PC-013 | Female | 46 | Rectum | No |
| PC-014 | Male | 50 | Rectum | Grandfather CRC, sister breast cancer |
| PC-015 | Female | 34 | Rectum | No |
| PC-016 | Male | 47 | Rectum | Grandfather CRC |
| PC-017 | Male | 40 | Rectum | No |
| PC-018 | Male | 49 | Sigmoid colon | No |
| PC-019 | Female | 43 | Rectum | No |
| PC-020 | Male | 47 | NA | No |
| PC-021 | Female | 39 | NA | No |

**Supplementary Table 2. List of rare high-impact variants.** Candidate genes and variants obtained after applying the prioritization algorithm. Chr: chromosome; gnomAD NFE=gnomAD 2.1.1 exome frequency in non-Finnish Europeans; SNP: dbSNP 144 identifier; NO: not observed; NA: not available; het: heterozygous; hom: homozygous; hem: hemizygous

| **Chr** | **Start** | **End** | **Gene** | **RefSeq and variant description (cDNA and protein)** | **gnomAD NFE** | **Observed frequency in discovery cohort** | **SNP** | **Samples (genotypes)** | **KEGG pathways** | **Gene/variant described in previous studies** |
| --- | --- | --- | --- | --- | --- | --- | --- | --- | --- | --- |
| 1 | 6,278,371 | 6,278,374 | *RNF207* | NM_207396:c.1675_1678del:p.Q559fs | 0.0001 | 0.0238 | rs769136472 | PC-009(het) |  |  |
| 1 | 6,637,086 | 6,637,086 | *TAS1R1* | NM_177540:c.788T>C:p.F263S | NO | 0.0714 | NA | PC-001(het)\|PC-004(het)\|PC-020(het) | |  |
| 1 | 25,166,429 | 25,166,429 | *CLIC4* | NM_013943:c.494delA:p.E165fs | NO | 0.0238 | NA | PC-018(het) |  |  |
| 1 | 40,422,771 | 40,422,773 | *MFSD2A* | NM_001136493:c.106_108del:p.36_36del | NO | 0.0238 | NA | PC-015(het) |  |  |
| 1 | 47,838,627 | 47,838,627 | *CMPK1* | NM_001136140:c.172G>A:p.E58K | NO | 0.0476 | rs72553946 | PC-004(hom) |  |  |
| 1 | 53,679,101 | 53,679,102 | *CPT2* | NM_000098:c.1811_1812del:p.P604fs | NO | 0.0238 | NA | PC-017(het) | hsa03320 (PPAR) |  |
| 1 | 54,476,045 | 54,476,045 | *LDLRAD1* | NM_001276392:c.262delC:p.H88fs | NO | 0.0238 | NA | PC-004(het) |  |  |
| 1 | 60,370,695 | 60,370,695 | *CYP2J2* | NM_000775:c.1039C>T:p.Q347X | NO | 0.0238 | rs774953352 | PC-020(het) |  |  |
| 1 | 84,880,273 | 84,880,273 | *DNASE2B* | NM_058248:c.184C>T:p.R62X | 0.0001 | 0.0238 | rs144779905 | PC-009(het) |  |  |
| 1 | 87,031,503 | 87,031,503 | *CLCA4* | NM_012128:c.755dupA:p.E252fs | 0.0005 | 0.0238 | rs781062541 | PC-011(het) |  |  |
| 1 | 100,316,615 | 100,316,616 | *AGL* | NM_000028:c.17_18del:p.Q6fs | 0.0001 | 0.0238 | rs113994127 | PC-017(het) |  |  |
| 1 | 144,930,790 | 144,930,790 | *PDE4DIP* | NM_001002811:c.919C>T:p.R307X | NO | 0.0238 | NA | PC-005(het) |  | Brea-Fernandez et al. 2017 |
| 1 | 144,955,288 | 144,955,288 | *PDE4DIP* | NM_001002810:c.146T>C:p.I49T | NO | 0.0952 | NA | PC-002(het)\|PC-003(het)\|PC-008(het)\|PC-009(het) | | Brea-Fernandez et al. 2017; Esteban-Jurado et al. 2015 |
| 1 | 145,518,259 | 145,518,259 | *PEX11B* | NM_001184795:c.319C>T:p.Q107X | NO | 0.0238 | NA | PC-014(het) |  |  |
| 1 | 146,765,298 | 146,765,298 | *CHD1L* | NM_004284:c.2398delT:p.L800X | NO | 0.0238 | NA | PC-012(het) |  | Chubb et al. Nat Comms 2016 |
| 1 | 147,092,506 | 147,092,506 | *BCL9* | NM_004326:c.2545T>C:p.S849P | NO | 0.0476 | rs782731004 | PC-021(hom) | hsa05200 (cancer) | deVoer et al. PloS Genet 2016 |
| 1 | 151,282,720 | 151,282,722 | *PI4KB* | NM_002651:c.955_957del:p.319_319del | NO | 0.0238 | rs769207623 | PC-004(het) |  |  |
| 1 | 153,907,306 | 153,907,306 | *DENND4B* | NM_014856:c.2702_2703insGCA:p.Q901delinsQQ | NO | 0.0238 | rs375088543 | PC-008(het) |  |  |
| 1 | 155,658,087 | 155,658,087 | *YY1AP1* | NM_001198903:c.169delG:p.E57fs | NO | 0.0238 | NA | PC-012(het) |  |  |
| 1 | 158,576,248 | 158,576,250 | *OR10Z1* | NM_001004478:c.20_22del:p.7_8del | NO | 0.0238 | rs763460505 | PC-002(het) |  |  |
| 1 | 173,503,660 | 173,503,662 | *SLC9C2* | NM_178527:c.1935_1937del:p.645_646del | NO | 0.0238 | NA | PC-014(het) |  |  |
| 1 | 212,619,171 | 212,619,171 | *NENF* | NM_013349:c.343-1G>A: | NO | 0.0238 | NA | PC-013(het) |  |  |
| 1 | 213,341,260 | 213,341,260 | *RPS6KC1* | NM_001136138:c.859delC:p.R287fs | NO | 0.0238 | rs773204726 | PC-021(het) |  |  |
| 10 | 50,678,568 | 50,678,568 | *ERCC6* | NM_000124:c.3437_3438insAAG:p.S1146delinsRS | NO | 0.0238 | NA | PC-013(het) | hsa03420 (NER) | Arora et al. 2015 Gastroenterology; Chubb et al. Nat Comms 2016 |
| 10 | 71,244,945 | 71,244,945 | *TSPAN15* | NM_012339:c.331G>A:p.V111M | 0.0019 | 0.0476 | rs150067289 | PC-006(hom) |  | deVoer et al. PloS Genet 2016 |
| 10 | 74,890,612 | 74,890,615 | *NUDT13* | NM_001283014:c.743_746del:p.L248fs | NO | 0.0238 | NA | PC-016(het) |  |  |
| 10 | 75,393,758 | 75,393,758 | *MYOZ1* | NM_021245:c.568C>T:p.R190X | NO | 0.0238 | rs763242972 | PC-008(het) |  |  |
| 10 | 78,316,976 | 78,316,976 | *C10orf11* | NM_032024:c.527G>A:p.G176E | 0.0002 | 0.0476 | rs75852090 | PC-011(het)\|PC-015(het) |  |  |
| 10 | 81,053,189 | 81,053,189 | *ZMIZ1* | NM_020338:c.1009C>G:p.R337G | NO | 0.0714 | NA | PC-006(het)\|PC-012(het)\|PC-019(het) | |  |
| 10 | 96,117,973 | 96,117,973 | *NOC3L* | NM_022451:c.218-1G>A: | 0.0001 | 0.0238 | rs755544153 | PC-008(het) |  |  |
| 10 | 99,968,178 | 99,968,179 | *R3HCC1L* | NM_001256620:c.307_308del:p.R103fs | NO | 0.0238 | NA | PC-003(het) |  |  |
| 10 | 106,014,981 | 106,014,981 | *GSTO1* | NM_001191002:c.95G>A:p.C32Y | 0.0006 | 0.0476 | rs45529437 | PC-012(het)\|PC-014(het) | hsa05200 (cancer) |  |
| 10 | 106,136,567 | 106,136,567 | *CFAP58* | NM_001008723:c.1091-2A>C: | NO | 0.0238 | rs201299517 | PC-009(het) |  |  |
| 10 | 118,630,712 | 118,630,712 | *ENO4* | NM_001242699:c.1125dupA:p.L375fs | NO | 0.0238 | NA | PC-008(het) |  | deVoer et al. PloS Genet 2016 |
| 11 | 865,582 | 865,582 | *TSPAN4* | NM_001025238:c.400C>A:p.L134I | NO | 0.0476 | NA | PC-005(hom) |  |  |
| 11 | 8,959,232 | 8,959,232 | *ASCL3* | NM_020646:c.476_477insAA:p.N159fs | 0.0009 | 0.0238 | rs564169914 | PC-013(het) |  | deVoer et al. PloS Genet 2016 |
| 11 | 10,582,187 | 10,582,189 | *LYVE1* | NM_006691:c.556_558del:p.186_186del | NO | 0.0238 | rs371513120 | PC-005(het) |  |  |
| 11 | 18,456,416 | 18,456,416 | *LDHC* | NM_002301:c.548delC:p.T183fs | NO | 0.0238 | rs750492944 | PC-009(het) |  |  |
| 11 | 45,945,703 | 45,945,703 | *GYLTL1B* | NM_001300722:c.276-2A>G: | NO | 0.0238 | rs759348831 | PC-001(het) |  |  |
| 11 | 47,647,265 | 47,647,265 | *MTCH2* | NM_001317232:c.683T>C:p.F228S | NO | 0.0238 | rs76666113 | PC-021(het) |  |  |
| 11 | 47,663,948 | 47,663,948 | *MTCH2* | NM_001317232:c.70G>A:p.V24M | NO | 0.0476 | rs186601647 | PC-005(het)\|PC-006(het)\|PC-011(het) | |  |
| 11 | 57,996,294 | 57,996,306 | *OR10Q1* | NM_001004471:c.42_54del:p.T14fs | 0.0002 | 0.0238 | rs771019506 | PC-017(het) |  |  |
| 11 | 61,106,875 | 61,106,875 | *TKFC* | NM_015533:c.454C>G:p.R152G | NO | 0.0238 | NA | PC-010(het) |  |  |
| 11 | 64,417,918 | 64,417,918 | *NRXN2* | NM_015080:c.3109+2T>G: | NO | 0.0238 | NA | PC-013(het) | hsa04514 (Cell adhesion) |  |
| 11 | 67,057,574 | 67,057,574 | *ANKRD13D* | NM_207354:c.116G>C:p.R39P | NO | 0.0714 | NA | PC-006(het) \|PC-015(het)\|PC-016(het) | |  |
| 11 | 67,814,983 | 67,814,983 | *TCIRG1* | NM_006053:c.601G>A:p.A201T | 0.0042 | 0.0476 | rs140963213 | PC-016(hom) |  | deVoer et al. PloS Genet 2016 |
| 11 | 103,027,174 | 103,027,174 | *DYNC2H1* | NM_001080463:c.3802delG:p.D1268fs | NO | 0.0238 | NA | PC-021(het) |  | deVoer et al. PloS Genet 2016; Esteban-Jurado et al 2015 |
| 11 | 123,886,809 | 123,886,811 | *OR10G4* | NM_001004462:c.528_530del:p.176_177del | NO | 0.0238 | rs546568617 | PC-005(het) |  |  |
| 11 | 134,241,378 | 134,241,379 | *GLB1L2* | NM_138342:c.1420_1421del:p.L474fs | NO | 0.0238 | NA | PC-015(het) |  |  |
| 12 | 550,058 | 550,059 | *CCDC77* | NM_001130148:c.1120_1121del:p.Y374fs | 0.0001 | 0.0238 | rs765908504 | PC-006(het) |  |  |
| 12 | 5,687,598 | 5,687,598 | *ANO2* | NM_001278596:c.2335C>T:p.R779W | 0.0005 | 0.0476 | rs189494523 | PC-003(het)\|PC-018(het) |  |  |
| 12 | 21,229,413 | 21,229,413 | *SLCO1B7* | NM_001009562:c.1634G>A:p.G545E | 0.0058 | 0.0714 | rs188817665 | PC-004(hom)\|PC-021(het) |  |  |
| 12 | 21,329,832 | 21,329,832 | *SLCO1B1* | NM_006446:c.481+1G>T: | 0.0001 | 0.0238 | rs77271279 | PC-005(het) |  |  |
| 12 | 52,884,701 | 52,884,701 | *KRT6A* | NM_005554:c.853C>T:p.Q285X | NO | 0.0238 | NA | PC-011(het) |  |  |
| 12 | 53,589,415 | 53,589,418 | *ITGB7* | NM_000889:c.1063_1066del:p.V355fs | NO | 0.0238 | NA | PC-008(het) | hsa04514 (Cell adhesion) | deVoer et al. PloS Genet 2016; Esteban-Jurado et al 2015 |
| 12 | 53,663,073 | 53,663,073 | *ESPL1* | NM_012291:c.347C>T:p.A116V | 0.0004 | 0.0476 | rs2364600 | PC-004(het)\|PC-015(het) |  | Arora et al. 2015 Gastroenterology |
| 12 | 54,350,333 | 54,350,333 | *HOXC12* | NM_173860:c.832G>A:p.A278T | 0.0003 | 0.0476 | rs76569642 | PC-005(het)\|PC-008(het) |  |  |
| 12 | 57,113,984 | 57,113,984 | *NACA* | NM_001113203:c.1329_1330insGTG:p.T444delinsVT | NO | 0.0238 | NA | PC-015(het) |  |  |
| 12 | 58,089,561 | 58,089,561 | *OS9* | NM_001261422:c.340-2A>G: | NO | 0.0238 | rs759837845 | PC-002(het) |  |  |
| 12 | 66,597,646 | 66,597,646 | *IRAK3* | NM_007199:c.289C>T:p.R97X | 0.0003 | 0.0238 | rs137909830 | PC-017(het) |  |  |
| 12 | 72,866,850 | 72,866,850 | *TRHDE* | NM_013381:c.1339delT:p.F447fs | NO | 0.0238 | NA | PC-006(het) |  |  |
| 12 | 95,531,447 | 95,531,447 | *FGD6* | NM_018351:c.2845C>T:p.Q949X | NO | 0.0238 | NA | PC-016(het) |  | deVoer et al. PloS Genet 2016 |
| 12 | 99,007,617 | 99,007,618 | *IKBIP* | NM_201612:c.798_799del:p.D266fs | 0.0001 | 0.0238 | rs762389884 | PC-018(het) |  |  |
| 12 | 104,370,739 | 104,370,739 | *TDG* | NM_003211:c.67C>T:p.Q23X | NO | 0.0238 | rs770094653 | PC-018(het) | hsa03410 (BER) | Chubb et al. Nat Comms 2016 |
| 12 | 113,875,765 | 113,875,765 | *SDSL* | NM_001304993:c.871C>A:p.L291I | 0.0001 | 0.0952 | rs146573098 | PC-004(het)\|PC-015(hom)\|PC-018(het) | |  |
| 12 | 122,359,410 | 122,359,410 | *WDR66* | NM_001178003:c.199_200insAAG:p.K67delinsKE | NO | 0.0714 | rs779416485 | PC-001(het)\|PC-012(het)\|PC-016(het) | |  |
| 12 | 123,276,570 | 123,276,570 | *CCDC62* | NM_201435:c.674delA:p.D225fs | NO | 0.0238 | rs766619523 | PC-005(het) |  |  |
| 12 | 123,425,400 | 123,425,400 | *ABCB9* | NM_019624:c.1394A>G:p.N465S | NO | 0.0476 | NA | PC-001(het)\|PC-013(het) |  |  |
| 12 | 123,920,692 | 123,920,692 | *RILPL2* | NM_145058:c.276delG:p.E92fs | NO | 0.0238 | NA | PC-017(het) |  |  |
| 13 | 32,709,040 | 32,709,040 | *FRY* | NM_023037:c.886-1G>A: | NO | 0.0238 | NA | PC-021(het) |  | deVoer et al. PloS Genet 2016 |
| 13 | 45,980,102 | 45,980,102 | *SLC25A30* | NM_001286806:c.70G>C:p.A24P | NO | 0.0476 | NA | PC-013(het)\|PC-019(het) |  |  |
| 13 | 96,511,913 | 96,511,913 | *UGGT2* | NM_020121:c.3757G>T:p.V1253F | NO | 0.0476 | NA | PC-005(het)\|PC-008(het)\|PC-009(het) | | deVoer et al. PloS Genet 2016 |
| 13 | 114,201,667 | 114,201,667 | *TMCO3* | NM_017905:c.1743delC:p.F581fs | NO | 0.0238 | NA | PC-017(het) |  |  |
| 14 | 20,528,398 | 20,528,399 | *OR4L1* | NM_001004717:c.195_196del:p.N65fs | 0.0001 | 0.0238 | rs33977614 | PC-015(het) |  |  |
| 14 | 23,420,768 | 23,420,768 | *HAUS4* | NM_001166269:c.461C>G:p.S154X | NO | 0.0238 | NA | PC-002(het) |  |  |
| 14 | 25,043,605 | 25,043,605 | *CTSG* | NM_001911:c.440G>A:p.W147X | NO | 0.0238 | rs747369127 | PC-020(het) |  | deVoer et al. PloS Genet 2016 |
| 14 | 101,195,359 | 101,195,359 | *DLK1* | NM_001317172:c.218A>T:p.Q73L | 0.0001 | 0.0476 | rs34686110 | PC-005(het)\|PC-006(het) |  |  |
| 14 | 105,722,803 | 105,722,803 | *BRF1* | NM_001242790:c.523delG:p.V175fs | NO | 0.0238 | rs764997703 | PC-018(het) |  | Bellido et al 2018 |
| 15 | 25,926,140 | 25,926,140 | *ATP10A* | NM_024490:c.3573+1G>A: | NO | 0.0238 | NA | PC-006(het) |  |  |
| 15 | 33,261,440 | 33,261,440 | *FMN1* | NM_001103184:c.1793A>G:p.K598R | 0.0005 | 0.0476 | rs61732708 | PC-004(het)\|PC-011(het) |  | Esteban-Jurado et al 2015 |
| 15 | 41,865,665 | 41,865,875 | *TYRO3* | NM_006293:c.2145_2145del:p.V715fs | NO | 0.0476 | NA | PC-017(het)\|PC-020(het) |  | deVoer et al. PloS Genet 2016 |
| 15 | 69,327,737 | 69,327,737 | *NOX5* | NM_001184779:c.815G>A:p.W272X | 0.0002 | 0.0238 | rs34406284 | PC-015(het) |  |  |
| 15 | 78,486,225 | 78,486,286 | *ACSBG1* | NM_001199377:c.518_530del:p.T173fs | NO | 0.0714 | NA | PC-003(het)\|PC-008(het)\|PC-017(het)\| | hsa03320 (PPAR) |  |
| 15 | 102,346,600 | 102,346,600 | *OR4F6* | NM_001005326:c.679dupA:p.Q226fs | NO | 0.0238 | rs767511892 | PC-008(het) |  | Chubb et al. Nat Comms 2016 |
| 16 | 21,108,722 | 21,108,722 | *DNAH3* | NM_017539:c.2619G>A:p.W873X | NO | 0.0238 | NA | PC-001(het) |  | deVoer et al. PloS Genet 2016 |
| 16 | 27,503,740 | 27,503,740 | *GTF3C1* | NM_001286242:c.3070C>T:p.R1024C | NO | 0.0476 | NA | PC-003(hom) |  |  |
| 16 | 29,755,702 | 29,755,702 | *C16orf54* | NM_175900:c.571C>T:p.R191W | 0.0009 | 0.0476 | rs35764197 | PC-005(hom) |  |  |
| 16 | 55,563,801 | 55,563,801 | *LPCAT2* | NM_017839:c.585dupA:p.R195fs | NO | 0.0238 | NA | PC-008(het) |  | deVoer et al. PloS Genet 2016 |
| 16 | 57,828,999 | 57,829,007 | *KIFC3* | NM_001130100:c.219_227del:p.73_76del | 0.0001 | 0.0238 | rs369201056 | PC-005(het) |  |  |
| 16 | 60,393,292 | 60,393,292 | *LOC729159* | NM_001282301:c.314dupC:p.A105fs | NO | 0.0238 | rs142375595 | PC-005(het) |  |  |
| 16 | 67,195,842 | 67,195,842 | *FBXL8* | NM_018378:c.152+2T>C: | 0.0004 | 0.0238 | rs117575136 | PC-018(het) |  |  |
| 16 | 74,951,908 | 74,951,908 | *WDR59* | NM_030581:c.887-2A>G: | NO | 0.0238 | NA | PC-004(het) |  | Esteban-Jurado et al 2015 |
| 17 | 3,100,937 | 3,100,947 | *OR1A2* | NM_012352:c.125_135del:p.N42fs | 0.0001 | 0.0238 | rs146311669 | PC-005(het) |  |  |
| 17 | 3,845,898 | 3,845,898 | *ATP2A3* | NM_005173:c.1545+2T>G: | NO | 0.0238 | NA | PC-015(het) |  |  |
| 17 | 4,859,892 | 4,859,892 | *ENO3* | NM_001193503:c.964dupT:p.G321fs | 0.0003 | 0.0238 | rs771868807 | PC-006(het) |  |  |
| 17 | 5,048,786 | 5,048,787 | *USP6* | NM_004505:c.2079_2080del:p.T693fs | 0.0005 | 0.0238 | rs560633910 | PC-017(het) |  | deVoer et al. PloS Genet 2016 |
| 17 | 7,291,937 | 7,291,955 | *TNK1* | NM_001251902:c.1705_1723del:p.P569fs | NO | 0.0238 | rs756087974 | PC-016(het) |  |  |
| 17 | 7,910,848 | 7,910,848 | *GUCY2D* | NM_000180:c.1566+2T>G: | NO | 0.0238 | NA | PC-005(het) |  |  |
| 17 | 10,304,362 | 10,304,362 | *MYH8* | NM_002472:c.3254+1G>A: | 0.0004 | 0.0238 | rs372740784 | PC-001(het) |  | Chubb et al. Nat Comms 2016; Esteban-Jurado et al 2015 |
| 17 | 10,398,511 | 10,398,512 | *MYH1* | NM_005963:c.5292_5293del:p.T1764fs | 0.0001 | 0.0238 | rs761653024 | PC-001(het) |  | deVoer et al. PloS Genet 2016; |
| 17 | 34,076,211 | 34,076,211 | *GAS2L2* | NM_139285:c.653C>T:p.T218M | NO | 0.0476 | NA | PC-011(het)\|PC-015(het) |  | Chubb et al. Nat Comms 2016 |
| 17 | 41,103,859 | 41,103,861 | *AARSD1,PTGES3L-AARSD1* | NM_001261434:c.1059_1061del:p.353_354del | NO | 0.0238 | rs768161954 | PC-005(het) |  |  |
| 17 | 42,476,539 | 42,476,544 | *GPATCH8* | NM_001304943:c.2667_2672del:p.889_891del | NO | 0.0238 | rs751873847 | PC-008(het) |  |  |
| 17 | 44,110,778 | 44,110,780 | *KANSL1* | NM_001193466:c.2713_2715del:p.905_905del | NO | 0.0238 | rs551968687 | PC-015(het) |  |  |
| 17 | 48,542,690 | 48,542,690 | *CHAD* | NM_001267:c.1049C>T:p.T350I | 0.0003 | 0.0476 | rs2231510 | PC-005(het)\|PC-015(het) | hsa04151 (Pi3K-Akt) | Esteban-Jurado et al 2015 |
| 17 | 48,625,669 | 48,625,669 | *SPATA20* | NM_001258372:c.103C>T:p.R35X | 0.0001 | 0.0238 | rs146830271 | PC-015(het) |  | deVoer et al. PloS Genet 2016; Esteban-Jurado et al 2015 |
| 17 | 56,348,226 | 56,348,226 | *MPO* | NM_000250:c.2031-2A>C: | 0.0071 | 0.0476 | rs35897051 | PC-019(hom) |  |  |
| 17 | 60,525,060 | 60,525,060 | *METTL2A* | NM_181725:c.917-2A>G: | NO | 0.0238 | NA | PC-004(het) |  |  |
| 17 | 72,916,507 | 72,916,507 | *USH1G* | NM_001282489:c.115G>A:p.E39K | 0.0005 | 0.0476 | rs111033466 | PC-015(hom) |  |  |
| 17 | 73,221,477 | 73,221,477 | *NUP85* | NM_001303276:c.635G>A:p.W212X | NO | 0.0238 | NA | PC-002(het) |  |  |
| 17 | 74,289,285 | 74,289,285 | *QRICH2* | NM_032134:c.1025delC:p.P342fs | NO | 0.0238 | rs773308093 | PC-020(het) |  | Smith et al. Hum Mut 2013 |
| 17 | 79,165,016 | 79,165,016 | *CEP131* | NM_001009811:c.2634G>C:p.E878D | NO | 0.0476 | rs143205584 | PC-015(hom) |  |  |
| 18 | 28,908,178 | 28,908,178 | *DSG1* | NM_001942:c.243G>C:p.Q81H | NO | 0.0476 | rs74368609 | PC-005(het)\|PC-015(het) |  |  |
| 18 | 47,364,152 | 47,364,152 | *MYO5B* | NM_001080467:c.4873G>T:p.E1625X | NO | 0.0238 | rs77401395 | PC-008(het) |  |  |
| 19 | 418,975 | 418,975 | *SHC2* | NM_012435:c.1702G>A:p.A568T | NO | 0.0476 | rs200038781 | PC-001(hom) |  |  |
| 19 | 1,271,387 | 1,271,449 | *CIRBP* | NM_001280:c.270_332del:p.90_111del | NO | 0.1190 | NA | PC-003(het)\|PC-010(het)\|PC-016(het)\|PC-019(het)\|PC-020(het) | |  |
| 19 | 1,615,703 | 1,615,703 | *TCF3* | NM_001136139:c.1567_1568insAGCTGAAGG:p.A523delinsELKA | NO | 0.0238 | rs781376654 | PC-011(het) | hsa05200 (cancer) |  |
| 19 | 5,867,421 | 5,867,423 | *FUT5* | NM_002034:c.314_316del:p.105_106del | NO | 0.0238 | NA | PC-002(het) |  |  |
| 19 | 9,089,838 | 9,089,838 | *MUC16* | NM_024690:c.1976_1977insGGC:p.A659delinsAA | 0.0004 | 0.0238 | rs145226180 | PC-015(het) |  |  |
| 19 | 10,221,667 | 10,221,672 | *PPAN,PPAN-P2RY11* | NM_001040664:c.1248_1253del:p.416_418del | NO | 0.0238 | rs749628554 | PC-014(het) |  |  |
| 19 | 11,326,148 | 11,326,148 | *DOCK6* | NM_020812:c.4022-1->A: | 0.0007 | 0.0476 | rs142339683 | PC-005(het)\|PC-015(het) |  | deVoer et al. PloS Genet 2016 |
| 19 | 15,514,491 | 15,514,491 | *AKAP8L* | NM_001291478:c.156_157insTATGGC:p.Q53delinsYGQ | 0.0007 | 0.0238 | rs757450061 | PC-009(het) |  | deVoer et al. PloS Genet 2016 |
| 19 | 34,912,496 | 34,912,496 | *PDCD2L* | NM_032346:c.870delC:p.S290fs | 0.0002 | 0.0238 | rs763351139 | PC-002(het) |  |  |
| 19 | 36,230,444 | 36,230,444 | *IGFLR1* | NM_024660:c.805C>G:p.P269A | 0.0003 | 0.0476 | rs138397992 | PC-019(het)\|PC-021(het) |  |  |
| 19 | 40,009,379 | 40,009,379 | *SELV* | NM_182704:c.835-1G>A: | NO | 0.0238 | NA | PC-012(het) |  |  |
| 19 | 44,081,294 | 44,081,294 | *PINLYP* | NM_001193621:c.23C>T:p.S8F | NO | 0.0238 | NA | PC-010(het) |  |  |
| 19 | 44,501,449 | 44,501,449 | *ZNF155* | NM_001260486:c.1441dupA:p.Q480fs | 0.0001 | 0.0238 | rs539072881 | PC-002(het) |  | Chubb et al. Nat Comms 2016 |
| 19 | 44,777,457 | 44,777,457 | *ZNF233* | NM_001207005:c.644delG:p.R215fs | NO | 0.0238 | NA | PC-020(het) |  |  |
| 19 | 49,685,865 | 49,685,865 | *TRPM4* | NM_001195227:c.1294G>A:p.A432T | 0.0005 | 0.0476 | rs201907325 | PC-005(het)\|PC-011(het) |  |  |
| 19 | 49,931,917 | 49,931,917 | *GFY* | NM_001195256:c.1399delT:p.F467fs | NO | 0.0238 | NA | PC-011(het) |  |  |
| 19 | 50,498,138 | 50,498,138 | *VRK3* | NM_001025778:c.653C>T:p.S218L | 0.0005 | 0.0476 | rs10410075 | PC-011(het)\|PC-018(het) |  |  |
| 19 | 50,512,606 | 50,512,606 | *VRK3* | NM_001308420:c.176C>T:p.S59F | 0.0005 | 0.0476 | rs2033262 | PC-011(het)\|PC-018(het) |  |  |
| 19 | 50,548,191 | 50,548,191 | *ZNF473* | NM_001308424:c.455C>T:p.T152M | 0.0005 | 0.0476 | rs16981705 | PC-011(het)\|PC-018(het) |  |  |
| 19 | 50,549,661 | 50,549,661 | *ZNF473* | NM_001308424:c.1925C>T:p.T642I | 0.0005 | 0.0476 | rs10424809 | PC-011(het)\|PC-018(het) |  |  |
| 2 | 21,224,812 | 21,224,814 | *APOB* | NM_000384:c.13480_13482del:p.4494_4494del | 0.0007 | 0.0238 | rs562574661 | PC-002(het) |  | deVoer et al. PloS Genet 2016 |
| 2 | 46,711,385 | 46,711,385 | *TMEM247* | NM_001145051:c.480T>G:p.F160L | NO | 0.0476 | NA | PC-009(het) \|PC-014(het) |  |  |
| 2 | 48,807,844 | 48,807,844 | *STON1,STON1-GTF2A1L* | NM_001198594:c.72delA:p.S24fs | NO | 0.0238 | rs779823083 | PC-012(het) |  |  |
| 2 | 101,624,332 | 101,624,332 | *TBC1D8* | NM_001102426:c.3373_3374insCTT:p.F1125delinsSF | 0.0005 | 0.0476 | rs150740812 | PC-015(hom) |  | deVoer et al. PloS Genet 2016 |
| 2 | 128,381,861 | 128,381,861 | *MYO7B* | NM_001080527:c.3935G>A:p.R1312Q | 0.0001 | 0.0476 | rs61743523 | PC-010(het)\|PC-011(het) |  |  |
| 2 | 225,266,039 | 225,266,040 | *FAM124B* | NM_001122779:c.446_447del:p.Y149fs | NO | 0.0238 | rs769064165 | PC-009(het) |  |  |
| 2 | 237,172,967 | 237,172,969 | *ASB18* | NM_212556:c.20_22del:p.7_8del | NO | 0.0238 | rs746608036 | PC-009(het) |  |  |
| 2 | 242,432,430 | 242,432,430 | *FARP2* | NM_014808:c.2874_2875insTTCT:p.L958fs | NO | 0.0238 | rs772078527 | PC-012(het) |  | deVoer et al. PloS Genet 2016 |
| 20 | 2,795,964 | 2,795,964 | *C20orf141* | NM_080739:c.134delC:p.A45fs | NO | 0.0238 | NA | PC-021(het) |  |  |
| 20 | 20,269,334 | 20,269,334 | *CFAP61* | NM_015585:c.2878C>T:p.R960X | NO | 0.0238 | rs114396838 | PC-015(het) |  |  |
| 20 | 24,944,580 | 24,944,580 | *APMAP* | NM_020531:c.1120C>T:p.R374W | 0.0003 | 0.0476 | rs147842490 | PC-008(het)\|PC-014(het) |  |  |
| 20 | 25,261,049 | 25,261,049 | *PYGB* | NM_002862:c.1239+1G>A: | NO | 0.0238 | NA | PC-004(het) |  |  |
| 20 | 25,657,232 | 25,657,232 | *ZNF337* | NM_001290261:c.692delC:p.T231fs | NO | 0.0238 | rs767394764 | PC-009(het) |  |  |
| 20 | 29,965,208 | 29,965,211 | *DEFB119* | NM_153289:c.93_96del:p.N31fs | 0.0002 | 0.0238 | rs374993061 | PC-014(het) |  | Smith et al. Hum Mut 2013 |
| 20 | 43,836,986 | 43,836,988 | *SEMG1* | NM_003007:c.1048_1050del:p.350_350del | NO | 0.0238 | rs757852428 | PC-009(het) |  |  |
| 20 | 57,897,451 | 57,897,452 | *EDN3* | NM_001302455:c.567_568del:p.T189fs | 0.0001 | 0.0238 | rs769685879 | PC-015(het) |  |  |
| 20 | 61,945,217 | 61,945,217 | *COL20A1* | NM_020882:c.2332G>C:p.A778P | NO | 0.0476 | rs768232368 | PC-001(hom) |  |  |
| 21 | 33,678,992 | 33,678,992 | *MRAP* | NM_178817:c.148G>A:p.V50M | 0.0002 | 0.0476 | rs75858661 | PC-005(hom) |  |  |
| 22 | 19,839,461 | 19,839,461 | *C22orf29* | NM_024627:c.324delT:p.D108fs | NO | 0.0238 | NA | PC-017(het) |  |  |
| 22 | 19,839,612 | 19,839,612 | *C22orf29* | NM_024627:c.173delT:p.V58fs | NO | 0.0238 | NA | PC-005(het) |  | Smith et al. Hum Mut 2013 |
| 22 | 22,869,006 | 22,869,006 | *ZNF280A* | NM_080740:c.948dupG:p.K317fs | NO | 0.0238 | rs750034126 | PC-008(het) |  |  |
| 22 | 42,422,781 | 42,422,822 | *WBP2NL* | NM_152613:c.526_567del:p.176_189del | NO | 0.0238 | rs753687229 | PC-018(het) |  |  |
| 22 | 42,522,724 | 42,522,724 | *CYP2D6* | NM_001025161:c.1193C>A:p.A398D | NO | 0.0476 | NA | PC-005(hom) |  |  |
| 22 | 46,653,363 | 46,653,364 | *PKDREJ* | NM_006071:c.5856_5857del:p.F1952fs | NO | 0.0238 | NA | PC-004(het) |  |  |
| 3 | 11,880,710 | 11,880,710 | *TAMM41* | NM_001284401:c.397C>T:p.R133X | NO | 0.0238 | NA | PC-017(het) |  |  |
| 3 | 39,135,499 | 39,135,501 | *WDR48* | NM_001303402:c.1634_1636del:p.545_546del | 0.0008 | 0.0238 | rs761080571 | PC-018(het) | hsa03460 (FA) | Chubb et al. Nat Comms 2016 |
| 3 | 40,503,521 | 40,503,529 | *RPL14* | NM_001034996:c.446_454del:p.149_152del | NO | 0.0238 | rs147295890 | PC-014(het) |  |  |
| 3 | 50,331,085 | 50,331,085 | *HYAL3* | NM_001200029:c.961dupG:p.D321fs | NO | 0.0238 | NA | PC-018(het) |  |  |
| 3 | 52,729,448 | 52,729,448 | *GLT8D1* | NM_018446:c.801delA:p.K267fs | NO | 0.0238 | rs776260345 | PC-020(het) |  |  |
| 3 | 57,321,953 | 57,321,953 | *ASB14* | NM_001142733:c.445G>T:p.E149X | 0.0001 | 0.0238 | rs376274765 | PC-015(het) |  |  |
| 3 | 58,178,455 | 58,178,455 | *DNASE1L3* | NM_001256560:c.786dupA:p.S263fs | NO | 0.0238 | NA | PC-004(het) |  |  |
| 3 | 58,520,669 | 58,520,676 | *ACOX2* | NM_003500:c.158_160del:p.53_54del | NO | 0.0238 | NA | PC-016(het) |  | deVoer et al. PloS Genet 2016 |
| 3 | 62,189,076 | 62,189,076 | *PTPRG* | NM_002841:c.1607C>T:p.T536M | 0.0008 | 0.0476 | rs149063170 | PC-013(hom) |  |  |
| 3 | 75,786,848 | 75,786,849 | *ZNF717* | NM_001128223:c.1925_1926del:p.T642fs | 0.0002 | 0.0238 | rs557324871 | PC-011(het) |  |  |
| 3 | 121,433,800 | 121,433,800 | *GOLGB1* | NM_001256488:c.1072G>A:p.E358K | NO | 0.0238 | NA | PC-004(het) |  | deVoer et al. PloS Genet 2016 |
| 3 | 148,895,767 | 148,895,767 | *CP* | NM_000096:c.2879-1G>T: | NO | 0.0238 | NA | PC-004(het) |  |  |
| 3 | 151,134,105 | 151,134,191 | *MED12L* | NM_053002:c.6198_6284del:p.2066_2095del | NO | 0.0238 | NA | PC-020(het) |  |  |
| 3 | 160,155,843 | 151,134,191 | *TRIM59* | NM_173084:c.1126_1129del:p.S376fs | NO | 0.0238 | NA | PC-015(het) |  |  |
| 3 | 184,075,148 | 151,134,191 | *CLCN2* | NM_001171088:c.766+2T>G: | NO | 0.0238 | NA | PC-020(het) |  |  |
| 3 | 186,959,345 | 151,134,191 | *MASP1* | NM_001879:c.1229-2A>G: | NO | 0.0238 | NA | PC-009(het) |  |  |
| 3 | 195,451,917 | 151,134,191 | *MUC20* | NM_001282506:c.443G>T:p.S148I | 0.0003 | 0.1667 | rs369019587 | PC-008(het)\|PC-012(het)\|PC-016(het)\|PC-017(het)\|PC-019(het)\|PC-020(het)\|PC-021(het) | | |
| 4 | 499,644 | 151,134,191 | *PIGG* | NM_001127178:c.498G>A:p.W166X | NO | 0.0238 | NA | PC-010(het) |  |  |
| 4 | 9,784,933 | 151,134,191 | *DRD5* | NM_000798:c.1280_1282del:p.427_428del | NO | 0.0238 | rs760013973 | PC-006(het) |  |  |
| 4 | 68,919,732 | 151,134,191 | *TMPRSS11F* | NM_207407:c.1212delA:p.V404fs | NO | 0.0238 | NA | PC-016(het) |  |  |
| 4 | 68,964,716 | 151,134,191 | *TMPRSS11F* | NM_207407:c.38_52del:p.13_18del | NO | 0.0238 | rs146311461 | PC-015(het) |  |  |
| 4 | 71,347,295 | 151,134,191 | *MUC7* | NM_152291:c.834_902del:p.278_301del | NO | 0.0238 | NA | PC-018(het) |  |  |
| 4 | 81,121,284 | 151,134,191 | *PRDM8* | NM_001099403:c.50C>G:p.A17G | NO | 0.0238 | NA | PC-016(het) |  |  |
| 4 | 87,686,643 | 151,134,191 | *PTPN13* | NM_080685:c.4258+2T>C | 0.0002 | 0.0238 | rs768524267 | PC-008(het) | hsa04210 (Apoptosis) | Esteban-Jurado et al 2015 |
| 4 | 106,155,921 | 151,134,191 | *TET2* | NM_001127208:c.822delC:p.I274fs | NO | 0.0238 | rs777145283 | PC-006(het) |  | Jansen et al. Familial Cancer 2019 |
| 4 | 111,482,686 | 151,134,191 | *ENPEP* | NM_001977:c.2846G>A:p.W949X | NO | 0.0238 | NA | PC-002(het) |  | deVoer et al. PloS Genet 2016; Chubb et al. Nat Comms 2016 |
| 4 | 113,539,372 | 151,134,191 | *ZGRF1* | NM_018392:c.1825_1826del:p.L609fs | NO | 0.0238 | NA | PC-019(het) |  |  |
| 4 | 141,313,416 | 151,134,191 | *CLGN* | NM_004362:c.1607_1608insCTT:p.L536delinsLL | NO | 0.0238 | rs373992784 | PC-004(het) |  | deVoer et al. PloS Genet 2016 |
| 4 | 165,962,334 | 151,134,191 | *TRIM60* | NM_152620:c.1110_1112del:p.370_371del | NO | 0.0238 | NA | PC-019(het) |  |  |
| 4 | 184,240,826 | 151,134,191 | *CLDN22* | NM_001111319:c.543_546del:p.C181fs | NO | 0.0238 | rs777582403 | PC-013(het) | hsa04514 (Cell adhesion) |  |
| 4 | 190,878,658 | 151,134,191 | *FRG1* | NM_004477:c.537+1G>A: | NO | 0.0238 | NA | PC-001(het) |  |  |
| 5 | 33,984,425 | 151,134,191 | *SLC45A2* | NM_001012509:c.264delC:p.V88fs | 0.0002 | 0.0238 | rs775387808 | PC-020(het) |  |  |
| 5 | 36,037,996 | 151,134,191 | *UGT3A2* | NM_001168316:c.1096C>T:p.R366X | NO | 0.0238 | rs147315371 | PC-016(het) |  |  |
| 5 | 37,351,350 | 151,134,191 | *NUP155* | NM_001278312:c.665A>G:p.D222G | NO | 0.0476 | NA | PC-008(het)\|PC-011(het) |  |  |
| 5 | 76,932,809 | 151,134,191 | *OTP* | NM_032109:c.284C>T:p.A95V | 0.0063 | 0.0714 | rs148662448 | PC-011(het)\|PC-013(hom) |  |  |
| 5 | 78,610,444 | 151,134,191 | *JMY* | NM_152405:c.2429_2464del:p.810_822del | 0.0001 | 0.0238 | rs761561656 | PC-002(het) |  |  |
| 5 | 98,239,527 | 151,134,191 | *CHD1* | NM_001270:c.339_341del:p.113_114del | NO | 0.0238 | rs755931057 | PC-005(het) |  | Richards et al 1999; Esteban-Jurado et al 2015 |
| 5 | 137,721,747 | 151,134,191 | *KDM3B* | NM_016604:c.817_819del:p.273_273del | 0.0001 | 0.0238 | rs756177253 | PC-009(het) |  | deVoer et al. PloS Genet 2016 |
| 5 | 139,909,206 | 151,134,191 | *ANKHD1,ANKHD1-EIF4EBP3* | NM_017747:c.6675C>A:p.N2225K | NO | 0.0476 | rs561754605 | PC-015(hom) |  |  |
| 5 | 145,557,223 | 151,134,191 | *LARS* | NM_020117:c.11_12del:p.R4fs | NO | 0.0238 | rs777302019 | PC-019(het) |  |  |
| 5 | 148,679,893 | 151,134,191 | *AFAP1L1* | NM_001146337:c.205G>A:p.V69M | NO | 0.0476 | rs780204672 | PC-009(het)\|PC-016(het) |  |  |
| 5 | 161,520,833 | 151,134,191 | *GABRG2* | NM_198904:c.108-1G>T: | NO | 0.0238 | NA | PC-012(het) |  |  |
| 6 | 18,222,194 | 151,134,191 | *KDM1B* | NM_153042:c.1744C>T:p.R582X | NO | 0.0238 | NA | PC-016(het) |  |  |
| 6 | 24,472,859 | 151,134,191 | *GPLD1* | NM_001503:c.494_496del:p.165_166del | NO | 0.0238 | NA | PC-021(het) |  |  |
| 6 | 32,166,434 | 151,134,191 | *NOTCH4* | NM_004557:c.4607_4609del:p.1536_1537del | 0.0001 | 0.0238 | NA | PC-005(het) | hsa05200 (cancer); hsa05206 (miRNAs) | deVoer et al. PloS Genet 2016 |
| 6 | 32,370,727 | 151,134,191 | *BTNL2* | NM_001304561:c.694delG:p.V232fs | 0.0002 | 0.0238 | NA | PC-005(het) |  |  |
| 6 | 36,238,322 | 151,134,191 | *PNPLA1* | NM_001145717:c.86C>G:p.A29G | NO | 0.0476 | NA | PC-012(het)\|PC-019(het) |  |  |
| 6 | 39,159,405 | 151,134,191 | *KCNK5* | NM_003740:c.761A>C:p.K254T | NO | 0.0476 | NA | PC-002(het)\|PC-004(het) |  |  |
| 6 | 41,304,030 | 151,134,191 | *NCR2* | NM_001199509:c.258delC:p.D86fs | NO | 0.0238 | rs750146756 | PC-003(het) |  |  |
| 6 | 54,173,613 | 151,134,191 | *TINAG* | NM_014464:c.265_266del:p.R89fs | 0.0001 | 0.0238 | rs533842810 | PC-016(het) |  |  |
| 6 | 90,573,079 | 151,134,191 | *CASP8AP2* | NM_001137667:c.1651_1653del:p.551_551del | NO | 0.0238 | NA | PC-015(het) |  |  |
| 6 | 107,390,889 | 151,134,191 | *BEND3* | NM_001080450:c.1504_1506del:p.502_502del | NO | 0.0238 | rs781904336 | PC-001(het) |  |  |
| 6 | 154,411,220 | 151,134,191 | *OPRM1* | NM_001285527:c.250C>T:p.R84X | 0.0001 | 0.0238 | rs200869983 | PC-001(het) |  |  |
| 6 | 159,129,424 | 151,134,191 | *SYTL3* | NM_001009991:c.516+1G>T: | NO | 0.0238 | rs766689145 | PC-001(het) |  |  |
| 7 | 20,795,056 | 151,134,191 | *ABCB5* | NM_178559:c.2248C>T:p.Q750X | NO | 0.0238 | rs146527949 | PC-005(het) |  | Chubb et al. Nat Comms 2016 |
| 7 | 21,779,202 | 151,134,191 | *DNAH11* | NM_001277115:c.7825delA:p.K2609fs | NO | 0.0238 | NA | PC-011(het) |  |  |
| 7 | 92,027,064 | 151,134,191 | *ANKIB1* | NM_019004:c.2423G>A:p.R808H | 0.0003 | 0.0476 | rs200132000 | PC-015(het)\|PC-016(het) |  | deVoer et al. PloS Genet 2016 |
| 7 | 117,227,865 | 151,134,191 | *CFTR* | NM_000492:c.1657C>T:p.R553X | 0.0001 | 0.0238 | rs74597325 | PC-004(het) |  | deVoer et al. PloS Genet 2016 |
| 7 | 126,249,461 | 151,134,191 | *GRM8* | NM_000845:c.1448_1449del:p.T483fs | NO | 0.0238 | NA | PC-014(het) |  |  |
| 7 | 143,747,823 | 151,134,191 | *OR2A5* | NM_012365:c.329_330del:p.T110fs | 0.0001 | 0.0238 | rs147003252 | PC-005(het) |  |  |
| 7 | 144,015,524 | 151,134,191 | *OR2A1,OR2A42* | NM_001001802:c.307_310del:p.C103fs | 0.0001 | 0.0476 | rs559439902 | PC-018(hom) |  |  |
| 7 | 149,484,830 | 151,134,191 | *SSPO* | NM_198455:c.3652delG:p.A1218fs | 0.0002 | 0.0238 | rs137977793 | PC-018(het) |  |  |
| 8 | 20,003,323 | 151,134,191 | *SLC18A1* | NM_001142324:c.1432_1433insCCACGAAGG:p.E478delinsATKE | NO | 0.0238 | NA | PC-018(het) |  |  |
| 8 | 56,698,896 | 151,134,191 | *TGS1* | NM_024831:c.439_444del:p.147_148del | 0.0002 | 0.0238 | rs565363610 | PC-017(het) |  | Chubb et al. Nat Comms 2016 |
| 8 | 86,245,776 | 151,134,191 | *CA1* | NM_001291968:c.89C>T:p.A30V | 0.0002 | 0.0476 | rs7821248 | PC-002(het)\|PC-005(het) |  |  |
| 8 | 91,054,977 | 151,134,191 | *DECR1* | NM_001359:c.688dupA:p.G229fs | NO | 0.0238 | NA | PC-021(het) |  |  |
| 8 | 94,772,206 | 151,134,191 | *TMEM67* | NM_001142301:c.148_149insTAAT:p.I50fs | NO | 0.0238 | rs148545964 | PC-005(het) |  |  |
| 8 | 95,680,236 | 151,134,191 | *ESRP1* | NM_001034915:c.991C>G:p.R331G | NO | 0.0952 | NA | PC-006(het)\|PC-010(het)\|PC-011(het)\|PC-013(het) | |  |
| 8 | 110,509,266 | 151,134,191 | *PKHD1L1* | NM_177531:c.10446G>A:p.W3482X | NO | 0.0238 | NA | PC-020(het) |  |  |
| 8 | 124,516,848 | 151,134,191 | *FBXO32* | NM_148177:c.539G>A:p.W180X | NO | 0.0238 | NA | PC-019(het) |  |  |
| 8 | 145,624,024 | 151,134,191 | *CPSF1* | NM_013291:c.1641_1643del:p.547_548del | NO | 0.0238 | NA | PC-005(het) |  |  |
| 9 | 35,813,128 | 151,134,191 | *HINT2* | NM_032593:c.415A>T:p.K139X | 0.0001 | 0.0238 | rs751566903 | PC-014(het) |  |  |
| 9 | 37,770,737 | 151,134,191 | *TRMT10B* | NM_144964:c.720+1->TA: | NO | 0.0238 | rs150866114 | PC-011(het) |  | Chubb et al. Nat Comms 2016 |
| 9 | 74,674,274 | 151,134,191 | *C9orf57* | NM_001128618:c.36_40del:p.S12fs | 0.0001 | 0.0238 | rs149346363 | PC-011(het) |  |  |
| 9 | 101,518,825 | 151,134,191 | *ANKS6* | NM_173551:c.2203C>G:p.P735A | 0.0002 | 0.0476 | rs79414550 | PC-019(het)\|PC-021(het) |  |  |
| 9 | 101,552,584 | 151,134,191 | *ANKS6* | NM_173551:c.664C>T:p.R222W | 0.0058 | 0.0476 | rs41283630 | PC-009(hom) |  |  |
| 9 | 107,456,810 | 151,134,191 | *OR13D1* | NM_001004484:c.108delA:p.R36fs | 0.0001 | 0.0238 | rs368902184 | PC-005(het) |  |  |
| 9 | 119,461,763 | 151,134,191 | *TRIM32* | NM_001099679:c.1742_1743del:p.M581fs | NO | 0.0238 | NA | PC-021(het) |  |  |
| 9 | 125,438,244 | 151,134,191 | *OR1L3* | NM_001005234:c.836T>C:p.V279A | 0.0022 | 0.0952 | rs74634130 | PC-005(het)\|PC-011(het)\|PC-018(hom) | |  |
| X | 2,951,253 | 151,134,191 | *ARSH* | NM_001011719:c.1516G>A:p.V506M | 0.0010 | 0.0667 | rs144189290 | PC-018(hom) |  |  |
| X | 9,935,531 | 151,134,191 | *CLDN34* | NM_001195081:c.135dupC:p.Y45fs | NO | 0.0333 | NA | PC-019(het) |  |  |
| X | 18,947,424 | 151,134,191 | *PHKA2* | NM_000292:c.1246G>A:p.G416R | 0.0001 | 0.0667 | rs16980929 | PC-011(hom) |  | deVoer et al. PloS Genet 2016 |
| X | 24,197,356 | 151,134,191 | *ZFX* | NM_001178084:c.115G>C:p.V39L | NO | 0.0667 | rs763554468 | PC-016(hom) |  |  |
| X | 32,717,387 | 151,134,191 | *DMD* | NM_000109:c.649A>G:p.K217E | NO | 0.0667 | NA | PC-012(hom) |  | deVoer et al. PloS Genet 2016 |
| X | 34,961,853 | 151,134,191 | *FAM47B* | NM_152631:c.905_976del:p.302_326del | NO | 0.0333 | NA | PC-020(het) |  |  |
| X | 36,371,757 | 151,134,191 | *CFAP47* | NM_001304548:c.8812C>T:p.P2938S | NO | 0.0333 | NA | PC-008(het) |  |  |
| X | 38,664,744 | 151,134,191 | *MID1IP1* | NM_001098791:c.545G>A:p.G182D | 0.0041 | 0.0667 | rs143008624 | PC-016(hom) |  |  |
| X | 47,072,582 | 151,134,191 | *UBA1* | NM_153280:c.2838+2T>G: | NO | 0.0667 | NA | PC-010(het)\|PC-012(het)\|PC-013(het) | | deVoer et al. PloS Genet 2016 |
| X | 47,082,672 | 151,134,191 | *CDK16* | NM_001170460:c.178C>T:p.P60S | 0.0018 | 0.0667 | rs17550472 | PC-005(hom) |  |  |
| X | 48,419,185 | 151,134,191 | *TBC1D25* | NM_002536:c.1889G>A:p.R630H | NO | 0.0667 | NA | PC-011(hom) |  |  |
| X | 48,419,242 | 151,134,191 | *TBC1D25* | NM_002536:c.1946G>A:p.R649H | 0.0002 | 0.0667 | NA | PC-002(hom) |  |  |
| X | 49,034,506 | 151,134,191 | *PRICKLE3* | NM_001307979:c.587C>T:p.T196M | 0.0003 | 0.0667 | NA | PC-014(hom) | hsa04310 (Wnt signaling) |  |
| X | 55,172,680 | 151,134,191 | *FAM104B* | NM_001166699:c.188C>G:p.P63R | NO | 0.0333 | rs111638770 | PC-018(het) |  |  |
| X | 67,941,995 | 151,134,191 | *STARD8* | NM_001142504:c.2326C>T:p.L776F | NO | 0.0667 | rs747609411 | PC-006(hom) |  |  |
| X | 85,403,731 | 151,134,191 | *DACH2* | NM_001139514:c.107C>T:p.P36L | 0.0017 | 0.0667 | rs147377892 | PC-012(hom) |  |  |
| X | 102,832,119 | 151,134,191 | *TCEAL4* | NM_001300901:c.162dupG:p.S54fs | NO | 0.0333 | rs201917819 | PC-004(het) |  |  |
| X | 105,189,924 | 151,134,191 | *NRK* | NM_198465:c.4120C>T:p.R1374X | NO | 0.0667 | rs768058273 | PC-006(hom) |  |  |
| X | 106,808,139 | 151,134,191 | *FRMPD3* | NM_032428:c.1238C>T:p.T413M | NO | 0.0667 | rs772636612 | PC-006(hom) |  |  |
| X | 108,718,478 | 151,134,191 | *GUCY2F* | NM_001522:c.688C>T:p.R230W | 0.0008 | 0.0667 | rs33973457 | PC-005(hom) |  | deVoer et al. PloS Genet 2016 |
| X | 118,257,548 | 151,134,191 | *KIAA1210* | NM_020721:c.478G>A:p.E160K | NO | 0.0667 | NA | PC-002(hom) |  |  |
| X | 131,188,749 | 151,134,191 | *STK26* | NM_001042452:c.133C>T:p.R45C | 0.0074 | 0.1000 | rs56044451 | PC-013(het)\|PC-018(hom) |  |  |
| X | 152,969,508 | 151,134,191 | *BCAP31* | NM_001139441:c.383C>T:p.T128M | 0.0025 | 0.0667 | NA | PC-018(hom) |  |  |
| X | 153,688,842 | 151,134,191 | *PLXNA3* | NM_017514:c.319C>T:p.R107C | 0.0004 | 0.0667 | NA | PC-012(hom) |  |  |
| Y | 14,887,426 | 151,134,191 | *USP9Y* | NM_004654:c.2353G>A:p.E785K | NO | 0.1667 | rs754430497 | PC-017(hem) |  |  |

**Supplementary Table 3. Enrichment analysis based on KEGG pathways.** P-values for Fisher´s exact test on the number of variants in the selected pathways compared to the totality of rare, high-impact variants found in the discovery cohort. None of the values is statistically significant.

| **Pathway** | **Variants** | **Fisher p-value** |
| --- | --- | --- |
| PPAR signaling | 2 | 0.734 |
| DNA repair | 3 | 1 |
| Pi3K.Akt | 1 | 1 |
| Apoptosis | 1 | 1 |
| Wnt | 1 | 0.507 |
| Cell adhesion | 3 | 1 |
| Pathways in cancer | 4 | 0.535 |
| miRNAs in cancer | 1 | 1 |
| TGF-ß | 0 | NA |
| Colorectal cancer | 0 | NA |
| DNA replication | 0 | NA |
| Hippo signaling | 0 | NA |
| MAPK signaling | 0 | NA |

**Supplementary Table 4. Gene Ontology enrichment pathways using PANTHER.** Enrichment was measured using Reactome pathways. None of the pathways remain statistically significant after multiple-testing correction using false discovery rate (FDR). We depict only the top 100 pathways based on nominal p-values.

| **Reactome pathways** | **Nominal p-value** | **FDR-corrected p-value** |
| --- | --- | --- |
| Glycogen breakdown (glycogenolysis) (R-HSA-70221) | 1.19E-03 | 1.00E+00 |
| TET1,2,3 and TDG demethylate DNA (R-HSA-5221030) | 2.05E-03 | 1.00E+00 |
| Ion channel transport (R-HSA-983712) | 2.10E-03 | 1.00E+00 |
| Diseases associated with O-glycosylation of proteins (R-HSA-3906995) | 2.13E-03 | 1.00E+00 |
| Defective GALNT12 causes colorectal cancer 1 (CRCS1) (R-HSA-5083636) | 2.17E-03 | 9.90E-01 |
| Defective GALNT3 causes familial hyperphosphatemic tumoral calcinosis (HFTC) (R-HSA-5083625) | 2.17E-03 | 8.25E-01 |
| Defective C1GALT1C1 causes Tn polyagglutination syndrome (TNPS) (R-HSA-5083632) | 2.47E-03 | 8.06E-01 |
| Metabolism of carbohydrates (R-HSA-71387) | 2.99E-03 | 8.55E-01 |
| Transport of small molecules (R-HSA-382551) | 4.54E-03 | 1.00E+00 |
| Termination of O-glycan biosynthesis (R-HSA-977068) | 4.84E-03 | 1.00E+00 |
| Glycogen metabolism (R-HSA-8982491) | 4.84E-03 | 1.00E+00 |
| Dectin-2 family (R-HSA-5621480) | 6.39E-03 | 1.00E+00 |
| Stimuli-sensing channels (R-HSA-2672351) | 1.06E-02 | 1.00E+00 |
| Hyaluronan uptake and degradation (R-HSA-2160916) | 1.17E-02 | 1.00E+00 |
| Apoptotic cleavage of cellular proteins (R-HSA-111465) | 1.19E-02 | 1.00E+00 |
| Transport of Mature mRNA Derived from an Intronless Transcript (R-HSA-159231) | 1.36E-02 | 1.00E+00 |
| O-linked glycosylation (R-HSA-5173105) | 1.40E-02 | 1.00E+00 |
| Transport of Mature mRNAs Derived from Intronless Transcripts (R-HSA-159234) | 1.45E-02 | 1.00E+00 |
| SEMA3A-Plexin repulsion signaling by inhibiting Integrin adhesion (R-HSA-399955) | 1.52E-02 | 1.00E+00 |
| Carnitine metabolism (R-HSA-200425) | 1.52E-02 | 1.00E+00 |
| Fatty acids (R-HSA-211935) | 1.70E-02 | 1.00E+00 |
| Unclassified (UNCLASSIFIED) | 1.88E-02 | 1.00E+00 |
| Disorders of transmembrane transporters (R-HSA-5619115) | 1.99E-02 | 1.00E+00 |
| Fatty acid metabolism (R-HSA-8978868) | 2.09E-02 | 1.00E+00 |
| Hyaluronan metabolism (R-HSA-2142845) | 2.11E-02 | 1.00E+00 |
| Pre-NOTCH Processing in Golgi (R-HSA-1912420) | 2.33E-02 | 1.00E+00 |
| Metabolism of Angiotensinogen to Angiotensins (R-HSA-2022377) | 2.33E-02 | 1.00E+00 |
| Defective SLCO1B1 causes hyperbilirubinemia, Rotor type (HBLRR) (R-HSA-5619110) | 2.36E-02 | 1.00E+00 |
| snRNP Assembly (R-HSA-191859) | 2.52E-02 | 1.00E+00 |
| Metabolism of non-coding RNA (R-HSA-194441) | 2.52E-02 | 1.00E+00 |
| Scavenging by Class A Receptors (R-HSA-3000480) | 2.55E-02 | 1.00E+00 |
| Apoptotic execution phase (R-HSA-75153) | 2.64E-02 | 1.00E+00 |
| ER Quality Control Compartment (ERQC) (R-HSA-901032) | 3.03E-02 | 1.00E+00 |
| SLC transporter disorders (R-HSA-5619102) | 3.16E-02 | 1.00E+00 |
| tRNA processing in the nucleus (R-HSA-6784531) | 3.18E-02 | 1.00E+00 |
| Diseases of glycosylation (R-HSA-3781865) | 3.46E-02 | 1.00E+00 |
| Defective CP causes aceruloplasminemia (ACERULOP) (R-HSA-5619060) | 3.53E-02 | 1.00E+00 |
| Defective SLC40A1 causes hemochromatosis 4 (HFE4) (macrophages) (R-HSA-5619049) | 3.53E-02 | 1.00E+00 |
| Events associated with phagocytolytic activity of PMN cells (R-HSA-8941413) | 3.53E-02 | 1.00E+00 |
| ABC-family proteins mediated transport (R-HSA-382556) | 3.79E-02 | 1.00E+00 |
| Xenobiotics (R-HSA-211981) | 3.80E-02 | 1.00E+00 |
| Post-translational protein modification (R-HSA-597592) | 4.21E-02 | 1.00E+00 |
| RNA Polymerase III Transcription Initiation From Type 2 Promoter (R-HSA-76066) | 4.35E-02 | 1.00E+00 |
| Calnexin/calreticulin cycle (R-HSA-901042) | 4.35E-02 | 1.00E+00 |
| HDMs demethylate histones (R-HSA-3214842) | 4.35E-02 | 1.00E+00 |
| Metabolism (R-HSA-1430728) | 4.39E-02 | 1.00E+00 |
| RNA Polymerase III Transcription Initiation From Type 1 Promoter (R-HSA-76061) | 4.64E-02 | 1.00E+00 |
| RHO GTPases regulate CFTR trafficking (R-HSA-5627083) | 4.67E-02 | 1.00E+00 |
| O-linked glycosylation of mucins (R-HSA-913709) | 4.74E-02 | 1.00E+00 |
| Processing of Capped Intronless Pre-mRNA (R-HSA-75067) | 4.93E-02 | 1.00E+00 |
| Interconversion of nucleotide di- and triphosphates (R-HSA-499943) | 4.93E-02 | 1.00E+00 |
| Glycolysis (R-HSA-70171) | 5.09E-02 | 1.00E+00 |
| Regulation of Glucokinase by Glucokinase Regulatory Protein (R-HSA-170822) | 5.23E-02 | 1.00E+00 |
| Defective TPR may confer susceptibility towards thyroid papillary carcinoma (TPC) (R-HSA-5619107) | 5.23E-02 | 1.00E+00 |
| NEP/NS2 Interacts with the Cellular Export Machinery (R-HSA-168333) | 5.23E-02 | 1.00E+00 |
| Transport of Ribonucleoproteins into the Host Nucleus (R-HSA-168271) | 5.23E-02 | 1.00E+00 |
| Intracellular signaling by second messengers (R-HSA-9006925) | 5.35E-02 | 1.00E+00 |
| Inactivation, recovery and regulation of the phototransduction cascade (R-HSA-2514859) | 5.53E-02 | 1.00E+00 |
| Export of Viral Ribonucleoproteins from Nucleus (R-HSA-168274) | 5.53E-02 | 1.00E+00 |
| LDL remodeling (R-HSA-8964041) | 5.81E-02 | 1.00E+00 |
| Scavenging by Class H Receptors (R-HSA-3000497) | 5.81E-02 | 1.00E+00 |
| Ficolins bind to repetitive carbohydrate structures on the target cell surface (R-HSA-2855086) | 5.81E-02 | 1.00E+00 |
| SUMOylation of DNA damage response and repair proteins (R-HSA-3108214) | 5.82E-02 | 1.00E+00 |
| The phototransduction cascade (R-HSA-2514856) | 5.85E-02 | 1.00E+00 |
| Nuclear import of Rev protein (R-HSA-180746) | 5.85E-02 | 1.00E+00 |
| Vpr-mediated nuclear import of PICs (R-HSA-180910) | 5.85E-02 | 1.00E+00 |
| Transport of the SLBP independent Mature mRNA (R-HSA-159227) | 6.16E-02 | 1.00E+00 |
| SUMOylation of SUMOylation proteins (R-HSA-4085377) | 6.16E-02 | 1.00E+00 |
| Rev-mediated nuclear export of HIV RNA (R-HSA-165054) | 6.16E-02 | 1.00E+00 |
| Transport of the SLBP Dependant Mature mRNA (R-HSA-159230) | 6.49E-02 | 1.00E+00 |
| Nuclear Pore Complex (NPC) Disassembly (R-HSA-3301854) | 6.49E-02 | 1.00E+00 |
| Interactions of Vpr with host cellular proteins (R-HSA-176033) | 6.82E-02 | 1.00E+00 |
| Interactions of Rev with host cellular proteins (R-HSA-177243) | 6.82E-02 | 1.00E+00 |
| Defective LFNG causes SCDO3 (R-HSA-5083630) | 6.93E-02 | 1.00E+00 |
| Threonine catabolism (R-HSA-8849175) | 6.93E-02 | 1.00E+00 |
| Chylomicron clearance (R-HSA-8964026) | 6.93E-02 | 1.00E+00 |
| Scavenging by Class B Receptors (R-HSA-3000471) | 6.93E-02 | 1.00E+00 |
| Dopamine receptors (R-HSA-390651) | 6.93E-02 | 1.00E+00 |
| Melanin biosynthesis (R-HSA-5662702) | 6.93E-02 | 1.00E+00 |
| Fructose catabolism (R-HSA-70350) | 6.93E-02 | 1.00E+00 |
| VLDL assembly (R-HSA-8866423) | 6.93E-02 | 1.00E+00 |
| Hemostasis (R-HSA-109582) | 7.03E-02 | 1.00E+00 |
| RNA Polymerase III Transcription Initiation (R-HSA-76046) | 7.15E-02 | 1.00E+00 |
| N-glycan trimming in the ER and Calnexin/Calreticulin cycle (R-HSA-532668) | 7.15E-02 | 1.00E+00 |
| SUMOylation of ubiquitinylation proteins (R-HSA-3232142) | 7.15E-02 | 1.00E+00 |
| ROS and RNS production in phagocytes (R-HSA-1222556) | 7.49E-02 | 1.00E+00 |
| Striated Muscle Contraction (R-HSA-390522) | 7.49E-02 | 1.00E+00 |
| Transport of Mature Transcript to Cytoplasm (R-HSA-72202) | 7.65E-02 | 1.00E+00 |
| Gene and protein expression by JAK-STAT signaling after Interleukin-12 stimulation (R-HSA-8950505) | 7.84E-02 | 1.00E+00 |
| Platelet activation, signaling and aggregation (R-HSA-76002) | 8.01E-02 | 1.00E+00 |
| VLDL clearance (R-HSA-8964046) | 8.04E-02 | 1.00E+00 |
| Scavenging by Class F Receptors (R-HSA-3000484) | 8.04E-02 | 1.00E+00 |
| Pre-NOTCH Processing in the Endoplasmic Reticulum (R-HSA-1912399) | 8.04E-02 | 1.00E+00 |
| Biosynthesis of maresin-like SPMs (R-HSA-9027307) | 8.04E-02 | 1.00E+00 |
| mitochondrial fatty acid beta-oxidation of unsaturated fatty acids (R-HSA-77288) | 8.04E-02 | 1.00E+00 |
| NS1 Mediated Effects on Host Pathways (R-HSA-168276) | 8.19E-02 | 1.00E+00 |
| Metabolism of lipids (R-HSA-556833) | 8.43E-02 | 1.00E+00 |
| RNA Polymerase III Transcription (R-HSA-74158) | 8.90E-02 | 1.00E+00 |
| Class C/3 (Metabotropic glutamate/pheromone receptors) (R-HSA-420499) | 8.90E-02 | 1.00E+00 |
| RNA Polymerase III Abortive And Retractive Initiation (R-HSA-749476) | 8.90E-02 | 1.00E+00 |

**Supplementary Table 5. MPG rare, high-impact variants in the 8 candidate genes.** Twenty three rare, high-impact variants were found on the 267 non-cancer control samples from the Spanish MPG reference dataset. No variants were found for *TDG*

| **Gene** | **Variant** | **avsnp147** |
| --- | --- | --- |
| *CHAD* | NM_001267:c.1019C>T:p.T340M | rs771331186 |
| *CHAD* | NM_001267:c.735C>A:p.Y245X | rs776363437 |
| *CHD1L* | NM_004284:c.2179C>A:p.P727T | rs149913688 |
| *CHD1L* | NM_004284:c.2497A>G:p.K833E | rs782015121 |
| *CHD1L* | NM_004284:c.200G>A:p.C67Y | rs782470880 |
| *ERCC6* | NM_000124:c.3061A>G:p.I1021V | rs41562713 |
| *ERCC6* | NM_000124:c.2645A>G:p.Y882C | rs116431130 |
| *ERCC6* | NM_000124:c.2390C>G:p.S797C | rs146043988 |
| *ERCC6* | NM_000124:c.3650T>G:p.F1217C | rs61760166 |
| *ITGB7* | NM_000889:c.1103T>C:p.V368A | rs200281008 |
| *ITGB7* | NM_000889:c.1274G>A:p.R425Q | rs201711140 |
| *ITGB7* | NM_000889:c.2365C>T:p.R789C | rs141610554 |
| *PTPN13* | NM_080685:c.3140A>G:p.E1047G | rs200688335 |
| *PTPN13* | NM_080685:c.2695A>G:p.R899G | rs746747827 |
| *PTPN13* | NM_080685:c.3518T>C:p.L1173P | rs754275962 |
| *PTPN13* | NM_080685:c.7337G>A:p.R2446H | rs182154840 |
| *PTPN13* | NM_080685:c.5543C>T:p.T1848I | rs773669996 |
| *PTPN13* | NM_080685:c.793C>T:p.R265C | rs760047283 |
| *SPATA20* | NM_022827:c.1135G>C:p.G379R | rs201867412 |
| *SPATA20* | NM_022827:c.1480C>T:p.R494W | rs548494887 |
| *SPATA20* | NM_022827:c.301C>T:p.P101S | rs573597278 |
| *TGS1* | NM_024831:c.167-1G>A | rs769323976 |
| *TGS1* | NM_024831:c.31G>C:p.E11Q | rs61754981 |

**Supplementary Table 6. Enrichment tests for rare, high-impact variants on the eight candidate genes.** Fisher´s exact tests were used to compare the frequencies of the variants in the study cohorts versus the MPG non-cancer Spanish reference dataset.

| **Gene** | **Cumulative alleles study cohorts** | **Frequency** | **Alleles MPG** | **Frequency in MPG** | **Fisher enrichment p-value** |
| --- | --- | --- | --- | --- | --- |
| *CHAD* | 5 | 0.005 | 2 | 0.003 | 1 |
| *CHD1L* | 6 | 0.006 | 3 | 0.005 | 1 |
| *ERCC6* | 2 | 0.002 | 4 | 0.007 | 0.193 |
| *ITGB7* | 2 | 0.002 | 3 | 0.005 | 0.352 |
| *PTPN13* | 4 | 0.004 | 6 | 0.011 | 0.18 |
| *SPATA20* | 3 | 0.003 | 3 | 0.005 | 0.431 |
| *TDG* | 3 | 0.003 | 0 | 0 | 0.556 |
| *TGS1* | 3 | 0.003 | 2 | 0.003 | 1 |

**Supplementary Table 7: T1 Burden test results of Class 2 variants for exome sequencing as in Chubb et al. 2016.** Class 2 variants are defined in the original paper as nonsense, frameshift, missense predicted to be damaging and splice donor/acceptor-site variants. Total number of cases was 1006 and controls was 1609

| **Gene** | **Cases** | **Controls** | **p-value** |
| --- | --- | --- | --- |
| *CHD1L* | 43 | 46 | 3.19E-03 |
| *TDG* | 8 | 4 | 1.60E-02 |
| *TGS1* | 33 | 41 | 2.08E-02 |
| *ERCC6* | 41 | 51 | 2.68E-02 |
| *SPATA20* | 14 | 22 | 3.09E-01 |
| *ITGB7* | 11 | 17 | 3.15E-01 |
| *CHAD* | 0 | 3 | 8.62E-01 |
| *PTPN13* | 80 | 171 | 8.73E-01 |

**Supplementary Table 8. Somatic mutation profiles for TCGA individuals carrying rare, high-impact variants in the candidate genes.** COSMIC v2 signatures 4, 7, 11, 22 & 24 are related to nucleotide-excision repair, whereas signature 3 is related to homologous recombination, and is typical of BRCA-driven tumours. In bold: patients under 50 at diagnosis; *: patient carrying the missense somatic variant p.(P677L) in the same gene. In gray: signatures related to nucleotide-excision repair. 5-mC: 5-methylcitosine; HR: homologous recombination; ROS: reactive oxygen species; BER: base-excision repair. "+ Updated as per COSMIC v3.1”. RefSeq: *CHD1L*: NM_004284; *ERCC6*: NM_000124; *ITGB7*: NM_000889; *PTPN13*: NM_080685; *SPATA20*: NM_022827

| **Signature** | **Proposed_Etiology** | **Predominant cancer types** | ***CHD1L*:**  **p.(R468W)** | ***CHD1L*:**  **p.(M383I)** | ***ERCC6*:**  **p.(F1437I)** | ***ERCC6*:**  **p.(R683Q)*** | ***ERCC6*:**  **p.(G601S)** | ***ERCC6*:**  **p.(L224F)** | ***ITGB7*:**  **p.(Y758fs)** | ***ITGB7*:**  **p.(Y753C)** | ***PTPN13*:**  **p.(R782X)** | ***PTPN13*:**  **p.(R817C)** | ***PTPN13*:**  **p.S348T** | ***PTPN13*:**  **p.Q2482X** |
| --- | --- | --- | --- | --- | --- | --- | --- | --- | --- | --- | --- | --- | --- | --- |
| 1 | Age (spontaneous deamination of 5-mC) | All | 0.539 | 0.114 | 0.143 | 0.189 | 0.25 | 0.305 | 0.121 | 0.44 | 0.235 | 0.307 | 0.314 | 0.229 |
| 2 | APOBEC | Cervical, bladder | 0.018 | 0.017 | 0.01 | 0.007 | 0.04 | 0.014 | 0.004 | 0 | 0.001 | 0.013 | 0.007 | 0 |
| 3 | HR | Breast, ovarian, pancreatic | 0.2 | 0.336 | 0.319 | 0.216 | 0.368 | 0.338 | 0.194 | 0.301 | 0.363 | 0.273 | 0.328 | 0.336 |
| 4 | Smoking | Head and neck, liver, lung, oesophageal | 0 | 0 | 0 | 0.076 | 0 | 0 | 0.011 | 0.005 | 0 | 0 | 0 | 0 |
| 5 | Unknown | All | 0 | 0.173 | 0.306 | 0.173 | 0 | 0.001 | 0 | 0 | 0 | 0 | 0 | 0.07 |
| 6 | Defective MMR | Colorectal, uterine | 0.017 | 0 | 0 | 0 | 0.022 | 0 | 0 | 0.03 | 0 | 0.028 | 0.015 | 0 |
| 7 | UV | Skin, head and neck | 0.018 | 0.036 | 0.076 | 0.022 | 0 | 0.017 | 0.003 | 0 | 0.027 | 0 | 0.034 | 0.031 |
| 8 | Unknown | Breast and medulloblastoma | 0 | 0.025 | 0 | 0.032 | 0.04 | 0.085 | 0.104 | 0 | 0 | 0 | 0.053 | 0 |
| 9 | POLH | Chronic lymphocytic leukaemias, malignant B-cell lymphomas | 0 | 0.025 | 0 | 0.013 | 0.035 | 0 | 0.032 | 0.063 | 0 | 0.004 | 0 | 0 |
| 10 | POLE | Colorectal, uterine | 0 | 0.003 | 0 | 0 | 0 | 0 | 0.016 | 0 | 0 | 0.018 | 0 | 0 |
| 11 | Alkylating | Melanoma, glioblastoma | 0.03 | 0 | 0 | 0 | 0.051 | 0.044 | 0 | 0.008 | 0 | 0.038 | 0 | 0 |
| 12 | Unknown | Liver | 0.017 | 0.174 | 0.001 | 0.131 | 0.112 | 0.101 | 0.235 | 0.072 | 0.28 | 0.21 | 0.135 | 0.165 |
| 13 | APOBEC | Cervical, bladder | 0 | 0 | 0 | 0.024 | 0 | 0 | 0 | 0.006 | 0 | 0.011 | 0.012 | 0.003 |
| 14 | Unknown | Uterine | 0 | 0 | 0 | 0 | 0 | 0 | 0 | 0 | 0 | 0 | 0 | 0 |
| 15 | Defective MMR | Stomach | 0.009 | 0 | 0.008 | 0 | 0.019 | 0 | 0 | 0.017 | 0 | 0.003 | 0.014 | 0.046 |
| 16 | Unknown | Liver | 0 | 0.085 | 0.108 | 0 | 0 | 0.048 | 0 | 0.027 | 0 | 0 | 0.033 | 0 |
| 17 | Unknown | Oesophagus, breast, liver, lung, B-cell lymphoma, stomach, melanoma | 0.009 | 0.004 | 0 | 0 | 0.008 | 0 | 0.013 | 0.002 | 0.027 | 0.013 | 0 | 0.049 |
| 18 | Damage by ROS+ | Neuroblastoma | 0.041 | 0 | 0.009 | 0.028 | 0 | 0.03 | 0.02 | 0 | 0.012 | 0.031 | 0.029 | 0 |
| 19 | Unknown | Pilocytic astrocytoma | 0 | 0 | 0 | 0 | 0 | 0 | 0 | 0.014 | 0 | 0 | 0 | 0 |
| 20 | Defective MMR | Stomach, breast | 0 | 0 | 0 | 0 | 0 | 0 | 0.045 | 0 | 0.013 | 0.03 | 0 | 0.043 |
| 21 | Defective MMR+ | Stomach | 0 | 0 | 0 | 0 | 0 | 0 | 0 | 0 | 0 | 0 | 0 | 0 |
| 22 | Aristolochic acid | Renal, liver | 0 | 0 | 0.007 | 0.008 | 0 | 0 | 0.023 | 0 | 0.02 | 0.02 | 0.005 | 0.022 |
| 23 | Unknown | Liver | 0.012 | 0 | 0 | 0 | 0 | 0 | 0 | 0 | 0 | 0 | 0 | 0 |
| 24 | Aflatoxin exposure | Liver | 0 | 0 | 0 | 0.001 | 0 | 0 | 0.054 | 0 | 0 | 0 | 0 | 0 |
| 25 | Chemotherapy treatment+ | Hodgkin lymphomas | 0.06 | 0 | 0 | 0 | 0 | 0 | 0 | 0 | 0 | 0 | 0 | 0 |
| 26 | Defective MMR | Breast, cervical, stomach, uterine | 0 | 0 | 0 | 0 | 0 | 0 | 0 | 0 | 0 | 0.001 | 0 | 0.002 |
| 27 | Possible sequencing artefacts+ | Kidney clear cell | 0.004 | 0.005 | 0 | 0.015 | 0 | 0 | 0.016 | 0.014 | 0 | 0 | 0 | 0 |
| 28 | Unknown | Stomach | 0.024 | 0.004 | 0.012 | 0.01 | 0.015 | 0.019 | 0 | 0 | 0 | 0 | 0.021 | 0 |
| 29 | Tobacco chewing | Gingivo-buccal oral squamous cell carcinoma | 0 | 0 | 0 | 0 | 0.039 | 0 | 0 | 0 | 0 | 0 | 0 | 0 |
| 30 | Defective BER due to *NTHL1* mutations+ | Breast | 0.004 | 0 | 0 | 0.057 | 0 | 0 | 0.108 | 0 | 0.022 | 0 | 0 | 0.005 |

**Supplementary Table 8.** Continued

| **Signature** | **Proposed_Etiology** | **Predominant cancer types** | ***PTPN13*:**  **p.G1420R** | ***PTPN13*:**  **p.F724L** | ***PTPN13*:**  **p.T1383M** | ***PTPN13*:**  **p.D2110G** | *PTPN13*:  p.R2371H | ***PTPN13*:**  **p.R2446H** | *PTPN13*:  p.E1047G | *SPATA20*:  p.R51X | *SPATA20*:  p.V596M |
| --- | --- | --- | --- | --- | --- | --- | --- | --- | --- | --- | --- |
| 1 | Age (spontaneous deamination of 5-mC) | All | 0.231 | 0.255 | 0.085 | 0.351 | 0.256 | 0.05 | 0.132 | 0.288 | 0.092 |
| 2 | APOBEC | Cervical, bladder | 0.009 | 0.006 | 0 | 0 | 0.031 | 0 | 0.01 | 0 | 0.005 |
| 3 | HR | Breast, ovarian, pancreatic | 0.28 | 0.168 | 0.449 | 0.047 | 0.097 | 0.275 | 0.208 | 0.248 | 0.611 |
| 4 | Smoking | Head and neck, liver, lung, oesophageal | 0 | 0.009 | 0.079 | 0 | 0.036 | 0.041 | 0 | 0 | 0 |
| 5 | Unknown | All | 0 | 0 | 0 | 0 | 0 | 0 | 0.451 | 0.086 | 0 |
| 6 | Defective MMR | Colorectal, uterine | 0 | 0.175 | 0 | 0 | 0.088 | 0 | 0 | 0.052 | 0 |
| 7 | UV | Skin, head and neck | 0.05 | 0.016 | 0.004 | 0.014 | 0.001 | 0.017 | 0.027 | 0.059 | 0.038 |
| 8 | Unknown | Breast and medulloblastoma | 0 | 0 | 0 | 0.092 | 0.11 | 0 | 0.034 | 0.11 | 0 |
| 9 | POLH | Chronic lymphocytic leukaemias, malignant B-cell lymphomas | 0 | 0 | 0 | 0 | 0 | 0 | 0 | 0.009 | 0 |
| 10 | POLE | Colorectal, uterine | 0.008 | 0 | 0 | 0.004 | 0 | 0 | 0 | 0 | 0 |
| 11 | Alkylating | Melanoma, glioblastoma | 0 | 0 | 0.007 | 0 | 0 | 0 | 0 | 0.005 | 0 |
| 12 | Unknown | Liver | 0.272 | 0.193 | 0.064 | 0.048 | 0.184 | 0.415 | 0.09 | 0.109 | 0.126 |
| 13 | APOBEC | Cervical, bladder | 0 | 0.015 | 0 | 0.061 | 0 | 0 | 0.02 | 0 | 0 |
| 14 | Unknown | Uterine | 0 | 0 | 0 | 0 | 0 | 0 | 0 | 0 | 0 |
| 15 | Defective MMR | Stomach | 0 | 0 | 0 | 0.211 | 0 | 0.005 | 0 | 0 | 0.017 |
| 16 | Unknown | Liver | 0 | 0.023 | 0 | 0 | 0 | 0 | 0 | 0 | 0 |
| 17 | Unknown | Oesophagus, breast, liver, lung, B-cell lymphoma, stomach, melanoma | 0.011 | 0.004 | 0.016 | 0 | 0.02 | 0.041 | 0 | 0 | 0.052 |
| 18 | Damage by ROS+ | Neuroblastoma | 0 | 0.062 | 0 | 0 | 0.039 | 0.039 | 0 | 0 | 0.003 |
| 19 | Unknown | Pilocytic astrocytoma | 0 | 0 | 0 | 0 | 0 | 0 | 0 | 0 | 0 |
| 20 | Defective MMR | Stomach, breast | 0.07 | 0 | 0.093 | 0 | 0 | 0.056 | 0.011 | 0 | 0 |
| 21 | Defective MMR+ | Stomach | 0 | 0 | 0.174 | 0 | 0 | 0 | 0 | 0 | 0 |
| 22 | Aristolochic acid | Renal, liver | 0.014 | 0.044 | 0 | 0 | 0.058 | 0 | 0.009 | 0.001 | 0 |
| 23 | Unknown | Liver | 0 | 0 | 0.019 | 0 | 0 | 0 | 0 | 0 | 0 |
| 24 | Aflatoxin exposure | Liver | 0.022 | 0 | 0 | 0 | 0 | 0.027 | 0 | 0 | 0 |
| 25 | Chemotherapy treatment+ | Hodgkin lymphomas | 0 | 0 | 0 | 0.119 | 0.023 | 0 | 0 | 0 | 0 |
| 26 | Defective MMR | Breast, cervical, stomach, uterine | 0 | 0 | 0 | 0 | 0 | 0 | 0 | 0 | 0 |
| 27 | Possible sequencing artefacts+ | Kidney clear cell | 0 | 0 | 0 | 0 | 0.037 | 0.007 | 0 | 0 | 0 |
| 28 | Unknown | Stomach | 0.025 | 0 | 0.01 | 0.053 | 0 | 0 | 0.008 | 0.032 | 0 |
| 29 | Tobacco chewing | Gingivo-buccal oral squamous cell carcinoma | 0.006 | 0 | 0 | 0 | 0 | 0 | 0 | 0 | 0.056 |
| 30 | Defective BER due to *NTHL1* mutations+ | Breast | 0 | 0.029 | 0 | 0 | 0.018 | 0.028 | 0 | 0 | 0 |

**Figure S1. TDG immunostaining of the sigmoid colon adenocarcinoma for the patient carrying the p.(Q23X) variant.** (A) Microscopic appearance of adenocarcinoma stained with haematoxylin and eosin. Both the adenocarcinoma (B) and the adjacent normal mucosa (C) were positive for anti-TDG antibody. Original magnification, X400.


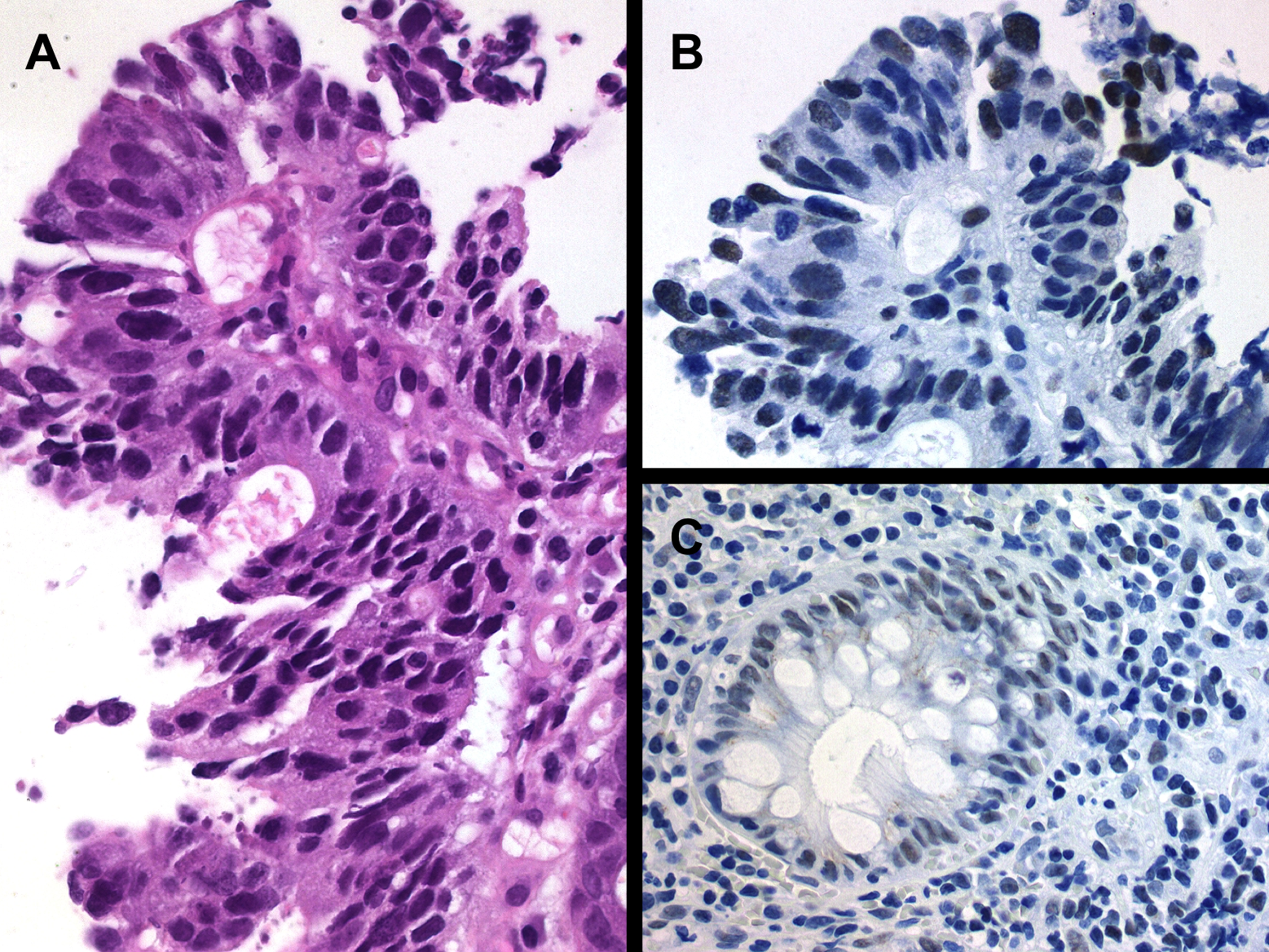


**Figure S2: Number of variants in all candidate genes from the discovery cohort found on TCGA samples.** We observe an overrepresentation of rare, high-impact variants in younger patients, albeit this trend is statistically not significant.
